# False-positive IRESes from *Hoxa9* and other genes resulting from errors in mammalian 5′ UTR annotations

**DOI:** 10.1073/pnas.2122170119

**Published:** 2022-08-29

**Authors:** Christina Akirtava, Gemma E. May, C. Joel McManus

**Affiliations:** ^a^Department of Biological Sciences, Carnegie Mellon University, Pittsburgh, PA 15213;; ^b^Computational Biology Department, Carnegie Mellon University, Pittsburgh, PA 15213

**Keywords:** Hox, IRES, UTR, translation, bicistronic

## Abstract

Hyperconserved genomic sequences have great promise for understanding core biological processes. It has been recently proposed that scores of hyperconserved 5′ untranslated regions (UTRs), also known as transcript leaders (hTLs), encode internal ribosome entry sites (IRESes) that drive cap-independent translation, in part, via interactions with ribosome expansion segments. However, the direct functional significance of such interactions has not yet been definitively demonstrated. We provide evidence that the putative IRESes previously reported in Hox gene hTLs are rarely included in transcript leaders. Instead, these regions function independently as transcriptional promoters. In addition, we find the proposed RNA structure of the putative *Hoxa9* IRES is not conserved. Instead, sequences previously shown to be essential for putative IRES activity encode a hyperconserved transcription factor binding site (E-box) that contributes to its promoter activity and is bound by several transcription factors, including *USF1* and *USF2*. Similar E-box sequences enhance the promoter activities of other putative *Hoxa* gene IRESes. Moreover, we provide evidence that the vast majority of hTLs with putative IRES activity overlap transcriptional promoters, enhancers, and 3′ splice sites that are most likely responsible for their reported IRES activities. These results argue strongly against recently reported widespread IRES-like activities from hTLs and contradict proposed interactions between ribosomal expansion segment ES9S and putative IRESes. Furthermore, our work underscores the importance of accurate transcript annotations, controls in bicistronic reporter assays, and the power of synthesizing publicly available data from multiple sources.

## Significance

Gene regulation is essential for mammalian development, and dysregulation is linked to many cancers. Scores of mammalian genes have been proposed to have hyperconserved 5′ transcript leaders that direct cap-independent translation via structured internal ribosome entry sites (IRESes). However, this model is based on bicistronic reporter assays, notoriously subject to false-positive results. We show that putative IRESes from *Hoxa9* and other mammalian genes generally encode transcriptional promoters and splice sites, and such features can be used to predict their activity in bicistronic reporters. These false-positive IRESes result from genome annotation errors and transcriptome complexity. Our work suggests these genes are expressed by canonical cap-dependent translation and highlights the importance of accurate transcript annotations for studying translational control.

As a critical step in gene expression, the translation of messenger RNA (mRNA) into protein is highly regulated. Eukaryotic translation is primarily controlled at the initiation stage, in which ribosomes identify start codons and begin synthesizing protein (1, 2). During proliferative growth, most mRNA translation initiates through a cap-dependent mechanism in which the 5′ 7-mG interacts with initiation factors to recruit a preinitiation complex (PIC) comprising the 40S small ribosomal subunit and multiple initiation factors. Once recruited, PICs scan directionally 5′ to 3′ until a start codon is recognized, the large ribosomal subunit is recruited, and translation commences. Under stress conditions, this cap-dependent translation is largely repressed due to inactivation of initiation factors. In such circumstances, ribosomes can be recruited to mRNA through cap-independent mechanisms, including internal ribosome entry sites (IRESes). IRESes are often found in viruses, as these pathogens often suppress cap-dependent translation of cellular RNAs to commandeer ribosomes for viral protein synthesis. IRESes have also been reported in cellular mRNA, although their roles in translation remain controversial (1, 2).

Several studies have coalesced on a surprising model in which hyperconserved transcript leaders (hTLs) include IRES-like sequences that drive cap-independent translation in specific cell types during development. These IRES-like elements were first proposed for mammalian *Hoxa* genes, based on the observation that the annotated mouse transcript leaders from several *Hoxa* genes drove expression in bicistronic luciferase assays, a classic test for cap-independent translation (3). It has also been proposed that ribosome expansion segment 9S (ES9S), a stem loop that protrudes from the ribosome, binds to a structured stem loop in the *Hoxa9* IRES-like sequence to recruit ribosomes to the *Hoxa9* transcript (4). Mammalian ES9S was also shown to bind G-rich motifs found in many mRNAs in vitro, which was proposed to drive cap-independent translation of many cellular transcripts (5).

The possibility of widespread cap-independent translation driven by interactions between IRESes and expansion segments is tantalizing. However, many previously reported IRESes in cellular mRNA have been fraught with controversy (6–8), especially when the sole evidence for such IRESes comes from bicistronic reporter assays. In these assays, a test IRES sequence is cloned between two luciferase open reading frames (ORFs), with the expectation that the downstream luciferase will only be expressed if the test sequence is an IRES. However, this assay is widely known to produce false positives resulting from monocistronic transcripts from transcriptional promoters or cryptic splicing in the IRES test sequence (9–14) (*SI Appendix*, Fig. S1). The bicistronic plasmid used in these studies (pRF) also has cryptic upstream promoters that generate unexpected monocistronic transcripts, which further complicates the interpretation of assay results (15) (*SI Appendix*, Fig. S1), It has also been noted that the Hox genes likely have much shorter transcript leaders than those used in bicistronic IRES assays (7). Furthermore, previous RNA interference (RNAi) control experiments suggested that putative *Hoxa* gene IRESes have independent promoter activity. While small interfering RNA (siRNA) targeting the upstream *Rluc* eliminated *Rluc* expression, ∼30% of *Fluc* expression resisted RNAi, indicating substantial monocistronic *Fluc* transcripts (3). However, the authors of the study inexplicably drew the opposite conclusion.

The proposed functional interaction between the *Hoxa9* P4 stem loop and ribosome ES9S is also problematic. Previous work found that sequences complementary to human ES9S did not support IRES activity (16). In addition, the proposed IRES RNA structures are inconsistent with functional assays. A cryoelectron microscopy (cryo-EM) structure model of this interaction appears to show the helices oriented as kissing stem loops. This structure most likely involves base pairing between nucleotides in the G-rich P4 loop and the C-rich loop of ES9S (*SI Appendix*, Fig. S2). However, mutations to the G-rich loop of P4 did not disrupt its apparent IRES activity (4, 5), suggesting that this proposed interaction is dispensable. Although some mutations that disrupt the P4 stem greatly reduced apparent IRES activity, compensatory mutations to restore P4 base pairing did not restore IRES function (4). Finally, a deletion of the 5′ half of the P4 stem loop did not disrupt IRES-like activity in the bicistronic reporter. As such, the authors could not rationalize how the P4/hES9S interaction visualized by cryo-EM related to bicistronic reporter expression (4). Together, these observations cast doubt on the model that ES9S binds to the *Hoxa9* P4 stem loop to drive cap-independent translation.

Recently, a high-throughput analysis defined a set of 589 hTLs with strong enrichment for genes involved in mammalian development (17). Hundreds of these hTLs were tested for IRES-like activities in bicistronic reporter assays, and 37% (90/241) drove substantial expression of the downstream luciferase cistron, suggesting that hTLs may frequently encode IRES-like functional elements. However, the possibility that these putative IRES activities may, instead, reflect functional promoter elements or cryptic 3′ splices sites was not directly investigated. While the authors showed that *Fluc*/*Rluc* protein and RNA ratios were not strongly correlated, this could result from variance in luciferase and RT-qPCR measurements (18, 19), especially considering that the pRF reporter plasmid expresses cryptic transcripts that would also be amplified (15). Indeed, previous work has cautioned against using RT-qPCR to normalize bicistronic reporter assays (20). Consequently, although they provide an alluring model for new modes of translational control during mammalian development, the authenticity of hTL IRES-like elements has not been conclusively established.

In this work, we investigate the possibility that putative IRES-like elements in mammalian hTLs instead encode transcriptional promoters and 3′ splice sites. We show that the putative *Hoxa9* IRES shows no signs of structural conservation and is rarely included in the gene’s transcript leader. Instead, sequences encoding putative IRESes from mouse *Hoxa* genes act as independent promoters. In addition, we demonstrate that a sequence previously identified as essential for *Hoxa9* IRES activity is a classical “E-box” site recognized by bHLH transcription factors. Putative IRESes from other *Hoxa* genes similarly have conserved E-box motifs that contribute to their promoter activities. Furthermore, the proposed IRES-like elements in the transcript leaders of *Chrdl1*, *Cnot3*, *Cryab*, and *Slc25a14* also have strong promoter activities. We also find that putative hTLs frequently overlap other functional elements, including protein CDSs, which could explain their conservation. Finally, we show that recently proposed IRES-like hTLs are overwhelmingly further enriched in annotated promoters, 3′ splice sites, and internal transcription initiation, and these elements can be used to accurately predict their reported IRES-like activities.

## Results

**Fig. 1.**
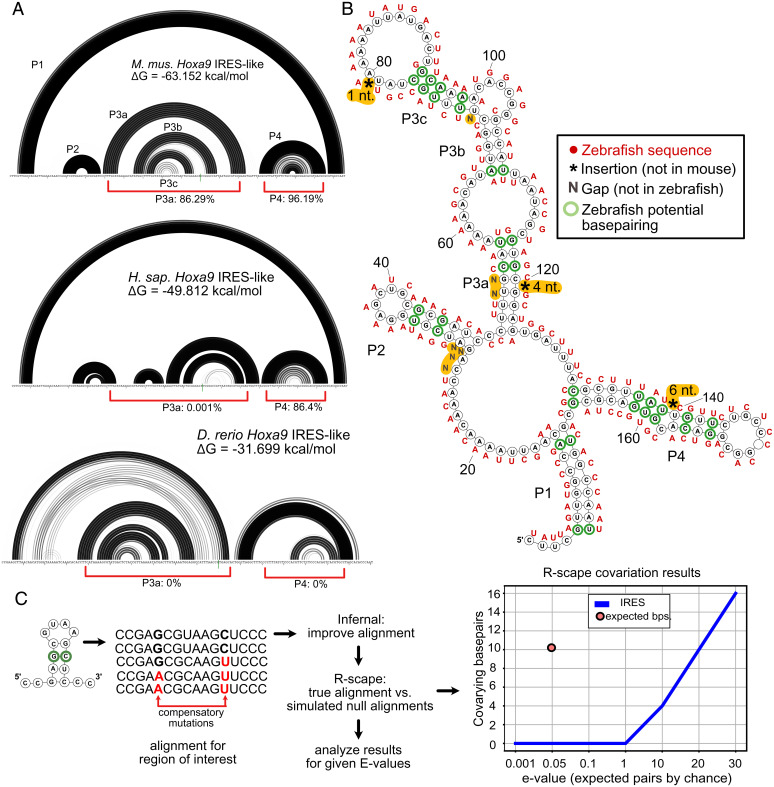
The proposed structure of the putative *Hoxa9* IRES element is not conserved. (*A*) Rainbow graphs depict the probability of base pairing for the *Hoxa9* IRES-like region from mouse (*Top*), human (*Middle*), and zebrafish (*Bottom*). Pairing regions (stems) are numbered as in ref. 3. Base pairing probabilities were determined using RNAstructure. The mouse model is highly consistent with the published model (3). Red brackets indicate the frequency of P3a and P4 helix formation in 10,000 predicted suboptimal structures. Human and mouse *Hoxa9* share P1 and P4, but lack P3, which was reported to be essential for IRES activity (3). Zebrafish *Hoxa9* does not share any structural similarity with mammalian homologs, despite driving bicistronic reporter activity (3). (*B*) Secondary structure model of mouse *Hoxa9* putative IRES region (3). Corresponding zebrafish sequences are shown in red. Most proposed base pairs are not conserved. Zebrafish has insertions (asterisks) and deletions (Ns) in the critical P3 and P4 elements. (*C*) Results of R-scape analysis of mutual information for the mouse *Hoxa9* putative IRES region using alignments from 208 mammals and 23 other vertebrates. The number of covarying sites (*y* axis) is given for different e-value cutoffs (*x* axis). Although the alignment has the power to detect ∼10 compensatory pairs (red point; Dataset S1), covarying base pairs are less common than expected by chance in the IRES-like element (blue line).

We first investigated the putative IRES region of *Hoxa9*, which has been called the paradigmatic example hTL (17). Many IRESes, including viral IRESes, fold into complex functional RNA structures. Previous studies reported a complex secondary structure for the *Hoxa9* IRES based on SHAPE probing (3). Two RNA base pairing regions (P3a and P4) were required for IRES activity (3, 4). The P4 stem loop was later shown to interact with ribosomal expansion segment ES9S in vitro (4), yet the P4 structure was not required for bicistronic reporter activity (4). Thus, the functional significance of the P4 region remains unclear. To further investigate the importance of the *Hoxa9* IRES structure, we examined its conservation. The putative IRES regions of both mouse and zebrafish *Hoxa9* were previously shown to drive bicistronic reporter expression in mouse cell culture (3). However, the predicted RNA structures of mouse, human, and zebrafish *Hoxa9* IRES regions differ substantially, such that the P3a domain is not predicted to form in the homologous human sequence, and neither P3a nor P4 are likely to form in zebrafish (Fig. 1 *A* and *B*). Furthermore, we found no evidence of significant structural covariation in RNA sequence alignments from 230 mammals (21) using Infernal (22) and R-scape (23) (Fig. 1*C*), despite having enough statistical power to detect such pairing (24) (Dataset S1). These results indicate that the proposed RNA structure of the *Hoxa9* IRES region, including domains previously reported to be essential for IRES activity, is not evolutionarily constrained.

**Fig. 2.**
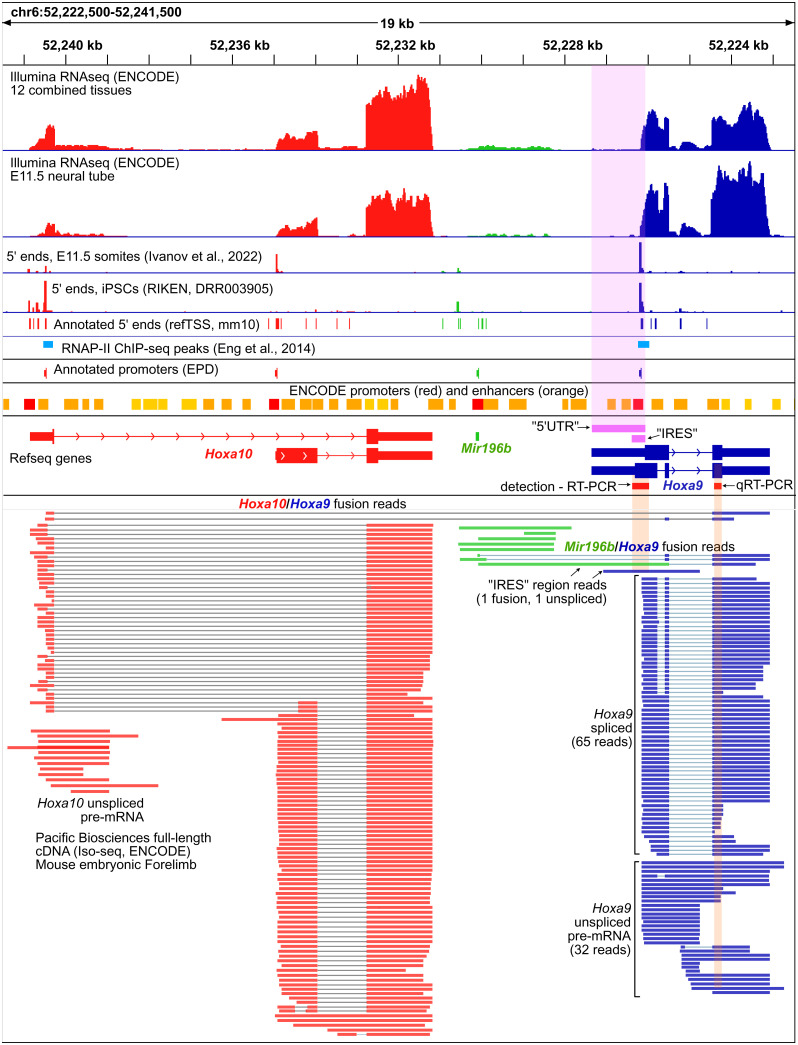
The putative extended 5′ UTR and IRES regions of mouse *Hoxa9* are not expressed at biologically meaningful levels. A genome browser view showing Refseq annotations of the mouse *Hoxa10*/*Mir196b*/*Hoxa9* in red, green, and blue, respectively, is shown. The putative TL(5′ UTR) and IRES regions are shown in pink. Promoters from the EPD and the ENCODE project are shown above the Refseq gene models. Illumina short-read (*Upper*) and PacBio full-length (*Lower*) RNA-seq data from the ENCODE consortium show negligible levels of RNA over the putative 5′ UTR and IRES regions. Similarly, two 5′ CAGE-seq studies [Ivanov et al. (56) and Abugessaisa et al. (53)] show no TSSs at the putative 5′ UTR, and a strong TSS peak downstream of the putative IRES. ChIP-seq peaks show RNAPII is found immediately downstream of EPD *Hoxa9* and *Hoxa10* promoters in mouse embryonic forelimbs [Eng et al. (29)]. PacBio RNA-seq detects *Hoxa9*/*a10* and *Hoxa9*/*Mir196b* fusion transcripts. Regions corresponding to the PCR amplicons used previously to detect (RT-PCR) the putative *Hoxa9* IRES and quantify (RT-qPCR) *Hoxa9* mRNA are shown in red. The *Hoxa9* RT-qPCR amplicon used for expression and polysome analysis is not specific to spliced *Hoxa9* mRNA, and can amplify fusion transcripts, unspliced transcripts, and truncated transcripts initiating at refTSS annotated start sites within *Hoxa9* introns.

To drive cap-independent translation, the mouse *Hoxa9* IRES must be included in its TL. Previous work noted that this may not be the case (7). Our evaluation of public RNA sequencing (RNA-seq) data indicates that the annotated TL of mouse *Hoxa9* used in previous studies shows little evidence of transcription in mouse tissues (Fig. 2 and *SI Appendix*, Fig. S3). In nearly all tissues analyzed, including embryonic neural tube, the tissue from which the IRES was first reported (3), ENCODE RNA-seq data show negligible levels of transcribed RNA in the upstream region of the annotated 5′ untranslated region (UTR). Instead, transcript levels sharply increase close to the *Hoxa9* start codon, immediately downstream of a strong transcription start site (TSS) annotated in the refTSS database. These short 5′ UTR isoforms are also supported by ENCODE long-read RNA-seq data from developing embryos (Fig. 2). Although the extended TL is partially supported by one long read, this appears to be an unspliced intron from *Hoxa10*/*Hoxa9* or *Mir196b*/*Hoxa9* fusion transcripts (Fig. 2). A very similar set of isoforms is supported by human RNA-seq data (*SI Appendix*, Fig. S3) (25–27) and also suggest the putative IRES region is excluded from translating mRNA (*SI Appendix*, Fig. S4) (28). Thus, the putative *Hoxa9* IRES is almost completely excluded from mouse and human *Hoxa9* transcripts.

**Fig. 3.**
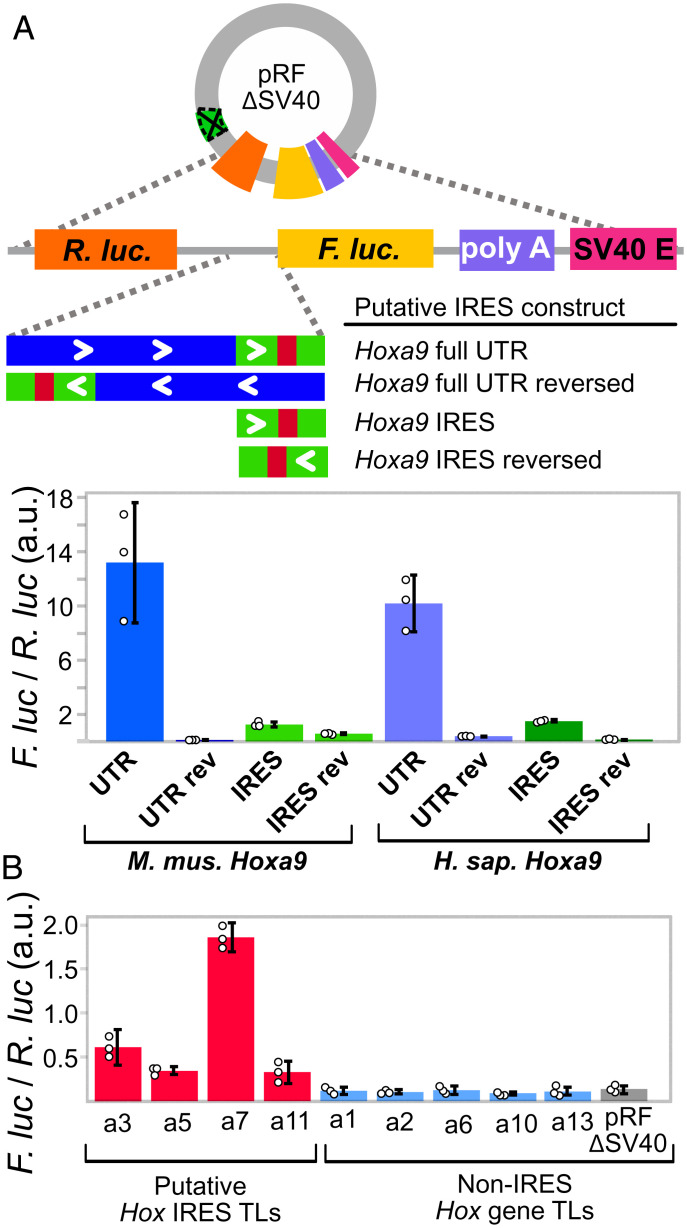
The putative IRES-like domains of *Hoxa9* and other Hox genes encode functional promoters. (*A*) The putative *Hoxa9* IRES is a promoter. The SV40 promoter was deleted from the pRF bicistronic vector. Putative IRES regions were cloned between *Renilla *luciferase (*Rluc*) and *Fluc* and tested for activity in C3H10T1/2 cells. Bar graphs show the *Fluc* to *Rluc* ratio indicating promoter activity from mouse and human *Hoxa9* regions. The extended UTR and IRES-like regions function as independent promoters in the forward orientation. (*B*) Putative IRES-like regions from other mouse *Hoxa* genes function as promoters. Annotated transcript leaders from each *Hoxa* gene were tested as in *A*. TLs containing putative IRES-like elements drove expression, while non-IRES TLs had background expression levels. Error bars show 95% CIs with *n* = 3.

As the putative *Hoxa9* IRES appears to be part of an intron from rare fusion transcripts, we examined an alternative hypothesis that the reported UTR region encodes functional DNA elements. Manual examination revealed the extended TL region overlapped two enhancers and a promoter annotated by the ENCODE consortium and the Eukaryotic Promoter Database (EPD) (21, 22) (Fig. 2). In addition, public chromatin immunoprecipitation (ChIP)-seq data from mouse embryos show RNAPII peaks just after the EPD promoter (29), consistent with promoter proximal pausing (30, 31) (Fig. 2). We tested this region for promoter activity in mouse C3H/10T1/2 embryonic mesenchymal cells, which were previously used to study the *Hoxa9* IRES (3). To most directly compare our results to previous IRES studies, we used a modified bicistronic reporter plasmid lacking the upstream SV40 promoter (pRF-ΔSV40) and cloned putative *Hoxa9* promoter regions between *Rluc* and *Fluc* (Fig. 3*A*). Strikingly, both the putative extended TL and putative IRES regions of mouse and human *Hoxa9* drove expression of Firefly luciferase (*Fluc*). Additionally, this expression was absent in reporters in which the putative IRES region was reversed (Fig. 3*A*). Finally, we used 5′ RACE to map *Fluc* TSSs used in the *Hoxa9* reporter and found they corresponded precisely to annotated mouse TSSs (*SI Appendix*, Fig. S5). These results show that the putative IRES region of *Hoxa9* encodes a functional promoter.

Based on bicistronic reporter assays, IRES-like activities were previously reported for the annotated TLs from other Hox genes, including *Hoxa3, Hoxa4, Hoxa5, Hoxa7,* and *Hoxa11*, while *Hoxa1*, *Hoxa2*, *Hoxa6*, *Hoxa10*, and *Hoxa13*, did not show activity (3, 17). We next investigated whether the IRES-like regions reported for these genes also have independent promoter activity. Remarkably, all of the previously reported IRES-like TLs we tested (*Hoxa3*, *Hoxa5*, *Hoxa7*, and *Hoxa11*) drove expression of *Fluc* independent of an upstream SV40 promoter, while the non-IRES TLs (*Hoxa1*, *Hoxa2*, *Hoxa6*, *Hoxa10*, and *Hoxa13*) had lower *Fluc*/*Rluc* ratios indistinguishable from background noise (Fig. 3*B*). Together, these results suggest the previously reported *Fluc* expression in bicistronic reporter plasmids containing upstream sequences from *Hoxa* genes was due to monocistronic *Fluc* transcripts driven by independent promoters, and not from bona fide IRESes.

**Fig. 4.**
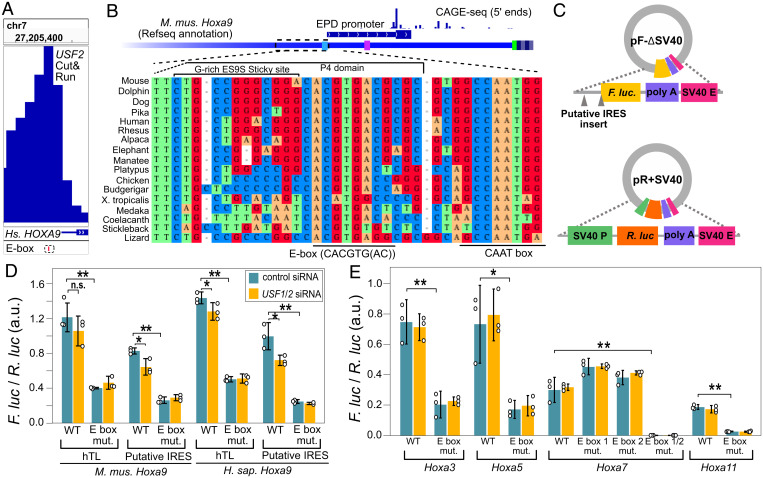
Putative IRES-like elements in *Hoxa* genes contain functional E-boxes recognized by *USF2*. (*A*) Genome browser view of CUT&RUN sequencing data shows the binding location of human *USF2* (37). The dashed box shows the location of a hyperconserved E-box motif (also see *SI Appendix*, Fig. S7). (*B*) Sequence alignment from diverse representative vertebrate genomes upstream of mouse *Hoxa9*, including the EPD promoter region. The diagram includes 5′ CAGE-seq data (53). The location of P4 domain sequences and the nonconserved G-rich ES9S interaction site are shown above, while the hyperconserved E-box and CAAT-box are noted below the alignment. The CAAT box and a TATA-like element are noted on the annotated *Hoxa9* transcript leader in light blue and magenta, respectively (*SI Appendix*, Fig. S8). (*C*) The *Fluc* and *Rluc* reporter genes were moved to two independent plasmids to test the functions of E-box elements. (*D*) Mutation of the E-box motif from mouse and human *Hoxa9* decreased expression in C3H10T1/2 cells. The siRNA codepletion of *USF1* and *USF2* decreased expression of wild-type (Welch’s one-tail *t* test, *P < 0.05; **P < 0.006), but not E-box mutant, reporters (n.s. signifies “not significant”). (*E*) Deletion of E-boxes from putative IRESes of *Hoxa3*, *Hoxa5*, *Hoxa7*, and *Hoxa11* decrease promoter activity. Single E-box mutations show slight increases in *Hoxa7* promoter activity, while the double mutation eliminated promoter function. Error bars show 95% CIs with *n* = 3.

We next considered previously identified critical sequences in the *Hoxa9* P4 region. Sequences in the highly conserved 3′ half of this region were previously shown to be required for bicistronic reporter activity and normal skeletal development (3, 4). Specifically, these were sensitive to mutations in the nucleotides underlined in “GACACGTGAC,” and similar sequences can be found in other putative *Hoxa* gene IRESes. Using FIMO (32) to search for transcription factor binding sites, we found this sequence matches E-box motifs recognized by at least 30 bHLH transcription factors, including *MYC*/*MAX*, *HES7*, and *ARNT2* (CACGTG) (33, 34), and *USF1*/*USF2*, *ARNTL*, and *TFE3* (CACGTGAC) (34–36). Recent work showed *USF2* binds upstream of human *Hoxa9*, and that codepletion of *USF1* and *USF2* decreases *Hoxa9* expression in human tissue culture cells (37) (Fig. 4*A*). Similarly, public mouse ChIP-seq data (38–51) show that *USF1*, *USF2*, and other bHLH factors bind to the *Hoxa9* E-box, and to E-box regions from other *Hoxa* genes (*SI Appendix*, Fig. S7). Remarkably, the *Hoxa9* E-box appears to be universally conserved in vertebrates, as nearly all species evaluated have the *USF1*/*USF2* motif. A CAAT box, considered a core promoter element, is universally conserved adjacent to the E-box, ∼60 nucleotides upstream of the major refTSS annotated TSS (Fig. 4*B* and *SI Appendix*, Fig. S8). Notably, the G-rich sequence in the loop region of the mouse *Hoxa9* P4 domain, which has a strong propensity to pair with mouse ES9S through a kissing stem loop interaction (*SI Appendix*, Fig. S2), is not similarly conserved in vertebrates. Thus, sequences reported to be essential for mouse *Hoxa9* IRES-like activity encode a deeply conserved transcription factor binding site known to drive expression of human *Hoxa9*.

We tested the hypothesis that these E-box motifs contribute to the promoter activity of Hox gene putative IRES regions. The promoter elements cloned between *Fluc* and *Rluc* in pRF-ΔSV40 plasmids appear to enhance spurious transcription of the upstream *Rluc* gene (15) to various extents (*SI Appendix*, Figs. S5 and S6; see *Discussion*), which could complicate interpretations of the effect of mutations on *Fluc* expression. Thus, we placed *Rluc* and *Fluc* on separate plasmids to assay the importance of E-box sites in mammalian *Hoxa* genes. Mutating the E-box motifs reduced the promoter activity of mouse *Hoxa3, Hoxa5, Hoxa7,* and *Hoxa11* and mouse and human *Hoxa9* IRES regions (Fig. 4 *D* and *E*; one-tail Welch’s *t* test *P* < 0.023). Furthermore, siRNA codepletion of mouse *USF1* and *USF2* (*SI Appendix*, Fig. S13) led to a statistically significant reduction in luciferase expression from wild-type mouse and human *Hoxa9* reporters, but not from reporters in which the E-box had been mutated (Fig. 4*D*; one-tail Welch’s *t* test *P* < 0.05). In contrast, *USF1*/*2* codepletion did not reduce expression from other mouse *Hoxa* putative IRESes (Fig. 4*E*), suggesting their E-boxes may be regulated primarily by other bHLH transcription factors. We conclude that the IRES-like regions of mouse *Hoxa* genes encode functional E-boxes. The function of these sequences as E-boxes explains their necessity for bicistronic reporter expression in previous studies of putative *Hoxa9* IRES activity.

**Fig. 5.**
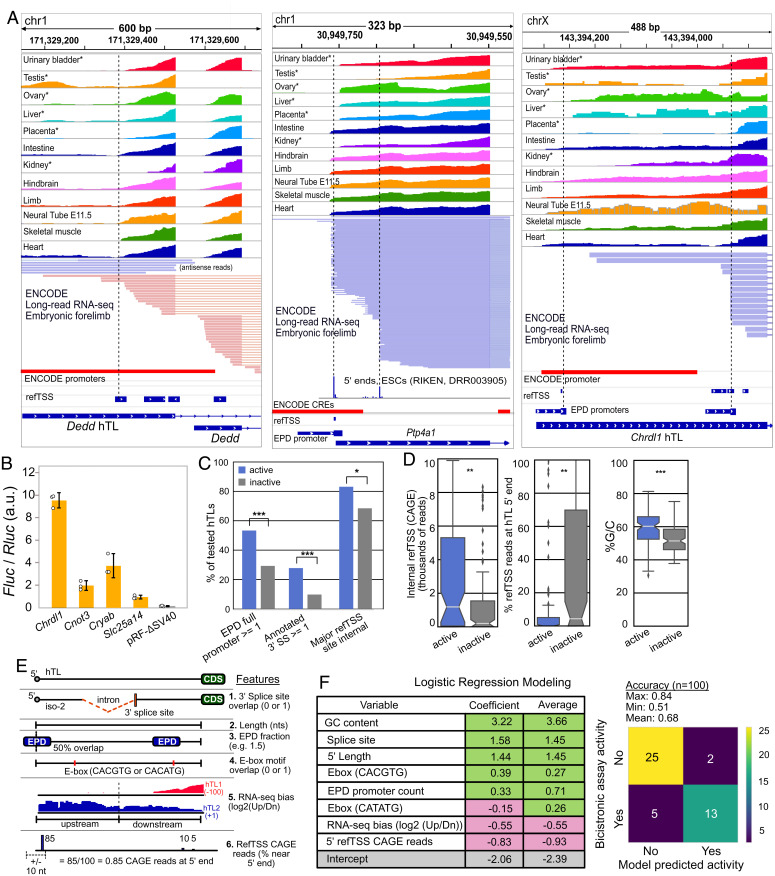
Putative IRES-like hTLs can be explained by promoters and 3′ splice sites due to 5′ UTR annotation errors. (*A*) Examples of promoter overlap commonly seen in putative IRES-like hTLs. Short-read (*Upper*) and long-read (*Lower*) RNA-seq data show transcription often initiates internally, coinciding with annotated promoters (ENCODE and EPD) and TSSs (refTSS). (*B*) The hTLs from four putative mouse IRESes have promoter activity in pRF-ΔSV40 transfected C3H10T1/2 cells. Error bars show 95% CIs with *n* = 3. (*C*) The hTLs with putative IRES-like activity are enriched in EPD promoters, 3′ splice sites, and major internal TSS sites (Χ^2^ tests). (*D*) IRES-active hTLs have significantly more internal CAGE 5′ reads, a lower fraction of TSS reads at annotated 5′ ends, and higher G/C content than IRES-inactive hTLs (Wilcoxon rank-sum tests). (*E*) Features for logistic regression modeling. RNA-seq bias is the ratio of reads in upstream and downstream hTL halves across GWIPs-viz RNA-seq datasets. refTSS CAGE reads are the percentage of 5′ end reads mapped near the annotated TSS (data from ref. 28). (*F*) Logistic regression modeling of IRES-like and non-IRES hTLs. Features associated with internal promoters guanine-cytosine (GC content, EPD promoter count fraction, E-boxes) and splice sites are positively correlated with bicistronic reporter expression, while features associated with full-length TLs (CAGE reads at annotated 5′ ends and RNA-seq 5′ end bias) are negatively correlated with bicistronic reporter activity. One hundred models were generated, with an average accuracy of 68%; *, **, and *** denote *P* < 0.05, 0.01, and 0.001, respectively.

Using the *Hoxa9* gene as a prototypical example of an hTL, a recent study identified 589 hTLs in the mouse genome. The authors tested over 200 of these elements in the bicistronic reporter system and reported 90 (37%) had IRES-like activity (17). Given the misannotation of *Hoxa* gene TLs, we next considered the possibility that these IRES-like hTLs may also be misannotated and encode functional promoters or 3′ splice sites, which both give false-positive results in bicistronic reporter assays. Evaluation of annotated promoter elements (51, 52), TSSs (53), annotated splice sites, and short- and long-read RNA-seq data (54) for TLs reported to have such IRES-like activities revealed the vast majority (85 of 90; 94%) have promoter and/or splicing elements. For example, the *Dedd* gene TL, reported to have the highest IRES-like activity, overlaps two ENCODE promoters and two refTSS sites. ENCODE short- and long-read RNA-seq data support transcription initiation inside the transcript leader, with almost no evidence of full-length hTL expression (Fig. 5*A*). Similarly, approximately one-third of transcripts from *Ptp4a1,* the second-strongest IRES-like hTL, initiate within the transcript leader. The IRES-like TL of *Chrdl1* also appears to be misannotated and encodes two EPD promoters with refTSS sites supporting internal transcription initiation (Fig. 5*A*). Other hTLs with reported IRES-like activity overlap known 3′ splice sites (*SI Appendix*, Fig. S9 *A* and *C*). We tested hTLs from four mouse genes that were reported to have IRES activity in C3H/10T1/2 cells (17)—*Chrdl1*, *Cryab*, *Cnot3*, and *Slc25a14*. All showed independent promoter activity in pRF-ΔSV40 (Fig. 5*B*). We also mapped the *Chrdl1*-driven *Fluc* TSS by 5′ RACE and found it matches its annotated EPD promoter and refTSS site (*SI Appendix*, Fig. S5). These results suggest that recently reported hTLs with IRES-like activity may also be false positives, such that their activities in bicistronic reporter assays result from monocistronic transcripts generated from internal promoters and 3′ splice sites rather than bona fide cap-independent translation.

We next systematically evaluated the 589 previously reported hTLs for potential misannotation due to overlapping promoters, enhancers, TSSs, 3′ splice sites, and protein coding sequences (CDSs), all of which could contribute to high conservation rates unrelated to translational control. Using combined promoter/enhancer sets, we find 93% of hTLs overlap ENCODE (509) and/or EPD (463) promoters (*SI Appendix*, Fig. S9*B*). Of those, 221 hTLs contain full-length EPD and 100 contain full-length ENCODE promoters. To further evaluate the accuracy of mouse TL annotations, we examined refTSS annotations and their underlying quantitative 5′-CAGE high-throughput sequencing data (53). Of the hTLs with public 5′ CAGE data, only 40% have an annotated refTSS site within 10 nucleotides of their annotated 5′ ends. Furthermore, 78% of all TSS-containing hTLs had the strongest CAGE peak within the hTL rather than near the 5′ end. The complexity of the mouse transcriptome further complicates conclusions about TL conservation, as 17% of hTLs overlap annotated 3′ splice sites and 43% of hTLs overlap annotated CDSs from alternative transcript isoforms (*SI Appendix*, Fig. S9*C*). Indeed, one-third of hTLs had CDS overlap covering at least 25% of their length, and one-fifth were at least 50% overlapped by annotated CDSs (*SI Appendix*, Fig. S9*D*). These data show that many recently reported hTLs are misannotated such that their high conservation rates may reflect evolutionary pressure to maintain promoters, enhancers, splice sites, and protein CDSs.

Our luciferase reporter assays show clear evidence that previously reported IRES-like elements result from transcriptional promoter activity. To further evaluate the potential for such false positives in recently reported hTLs, we compared the frequencies of promoters and splice sites, and the distribution of 5′ CAGE reads in hTLs that were reported to be “active” (90) and “nonactive” (133) in the bicistronic assay. Active hTLs are 1.7-fold more likely than nonactive hTLs to overlap at least one complete EPD promoter (53% vs. 29%; χ^2^ test *P* = 0.0002107; Fig. 5*C*). Similarly, annotated 3′ splice sites were 2.8-fold enriched in active, as compared to nonactive, hTLs (28% vs. 10%; χ^2^ test *P* = 0.000451; Fig. 5*C*). Furthermore, ∼83% of active TLs have their strongest CAGE peak within the TL compared to 68% for the nonactive TLs (χ^2^ test *P* = 0.02009; Fig. 5*C*). Finally, active hTLs have drastically more CAGE reads at internal refTSS sites (*P* = 0.00144), a lower fraction of CAGE reads near their annotated 5′ ends (*P* = 0.00173), and higher GC content (*P* = 2 × 10^−6^), which is characteristic of promoter elements (Fig. 5*D*) (55). The enrichment of promoters, splice sites, and internal 5′ CAGE reads in bicistronic active hTLs suggests that these elements generally drive bicistronic reporter expression through the creation of monocistronic *Fluc* transcripts, rather than by cap-independent IRES-like activities.

If internal promoters and splice sites are responsible for the reported IRES-like activities in hTLs, we reasoned that such features could be used to predict their activities. To test this, we used logistic regression with these features to model their activity in the bicistronic reporter assay (Fig. 5 *E* and *F*; see *Methods*). Strikingly, this approach generated models that were, on average, 68% accurate at predicting IRES-like activity (maximum accuracy 84%). Features associated with promoters (GC content, EPD promoter counts, E-boxes) and 3′ splice sites were positively correlated with bicistronic active hTLs, while those reflecting high levels of full-length hTL transcription (5′ refTSS fraction and 5′ RNA-seq bias) were associated with inactive hTLs. The ability to accurately predict bicistronic assay activity from these genomic features strongly supports the conclusion that such activities are false positives, inconsistent with their putative functions as IRES-like elements driving cap-independent translation.

## Discussion

The vast majority of mRNAs are believed to undergo cap-dependent translation in rapidly dividing cells, while cap-independent mechanisms, including IRESes, are used primarily during cell stress (2). Over the last decade, multiple studies have coalesced on an intriguing model proposing key developmental genes are regulated by cap-independent translation driven by IRES-like sequences and structures in hyperconserved 5′ transcript leaders (hTLs). However, this model was founded on bicistronic reporter assays, which are subject to common false-positive results due to cryptic promoter and splicing activities. Here, we examined previously reported hTLs with putative IRES-like activities in *Hoxa9* and other genes. We found that many putative IRES regions are rarely included in transcript leaders. In addition, the putative IRESes that are transcribed are enriched in internal promoters and/or 3′ splice sites known to cause false positives in bicistronic reporter assays. Consistent with this, a concurrent independent study found much shorter 5′ UTRs in *Hox* genes and showed the putative *Hoxa9* IRES has promoter activity in tissue culture cells (56). Furthermore, we successfully predicted putative IRES-like activity using known annotated promoters, 3′ splice sites, CAGE-seq 5′ end data, and public RNA-seq data. Finally, we find that promoters, splice sites, enhancers, and even protein CDSs overlap hTLs, which may explain their sequence conservation. Our results provide conventional explanations for unconventional results from previous studies, requiring a reevaluation of the proposal that these TLs drive cap-independent translation.

We found the putative *Hoxa9* IRES has only trace evidence of expression in mouse RNA-seq data and instead encodes a functional promoter. Consistent with this, our R-scape analysis, made possible by recently published mammalian genome sequences (21), indicates the proposed RNA structure of the *Hoxa9* IRES (3) is not constrained by evolution. Instead, the P4 region encodes a hyperconserved E-box motif recognized by *USF1*/*USF2* whose mutation drastically decreases promoter activity. Three recently reported mutations to this E-box, M3, M5, and M8 (4), modified one, four, and three nucleotides, respectively, and decreased expression in the bicistronic reporter assay, as expected for loss of a transcription factor binding site. Notably, the effects of these mutations corresponded to the number of nucleotides modified, as M3 had a smaller effect than M5 and M8. However, the M5 mutation also appeared to shift *Hoxa9* mRNA from the polysome toward the monosome (4). While this may seem to support IRES-like elements, it can also be explained by promoter activity. Mutating the E-box likely decreases the production of the natural 83-nt TL isoform, such that spurious longer transcripts, unspliced transcripts, and *Hoxa9*/*a10* fusion transcripts (Fig. 2) make up a larger fraction of *Hoxa9* mRNA in polysome gradients. These longer transcripts include up to 14 upstream ORFs (uORFs) and would not be translatable but would be detected using the RT-qPCR primers from these studies, which are not specific to spliced, mature *Hoxa9* mRNA (Fig. 2). Similar reasoning could explain how deletion of the putative IRES region appeared to alter *Hoxa9* polysome association in mouse embryos based on RT-qPCR (3). Thus, the intrinsic promoter activity we observed in *Hoxa9* genes provides a conventional explanation for the effects of these mutations, especially since the putative IRES is present in only ∼1% of mRNA transcripts in vivo (Fig. 2) (56).

A recent study also proposed the *Hoxa9* P4 stem loop recruits translation PICs through interactions with ribosomal expansion segment ES9S (4). However, these assays were performed at 4 °C and may not be physiologically relevant. Indeed, the P4 domain and ES9S have the potential to form kissing loops with nine G–C and one G–U base pairs, which appears consistent with a published cryo-EM structure (*SI Appendix*, Fig. S2) (4). This interaction has a predicted free energy of −15.94 kcal/mol (RNAcofold) (57) and would thus be very stable under cryo-EM and affinity purification conditions. Notably, neither the P4 structure nor the G-rich stretch of *Hoxa9* was required for IRES-like activity (4), and neither is evolutionarily conserved (Figs. 1 and 4). Because the putative IRES structure is not conserved, and is rarely, if ever, expressed as a transcript leader (Fig. 2 and *SI Appendix*, Figs. S3 and S4) (56), our results contradict the notion that mammalian ES9S recruits PICs to *Hoxa9* for cap-independent translation. A transcriptome-wide screen reported that ES9S similarly binds to mouse mRNA fragments with G-rich motifs, several of which were reported to have IRES-like activity, using the bicistronic reporter system (5). However, these putative IRESes often overlap promoter elements (*SI Appendix*, Fig. S10), and no controls were performed to test for false positives from monocistronic *Fluc*. Consequently, we propose that the interactions observed in vitro between the ES9S and G-rich mRNA are coincidental associations stabilized by low temperature.

The TLs of *Hoxa3*, *Hoxa4*, *Hoxa5*, *Hoxa7*, and *Hoxa11* were also previously reported to have IRES activity, based on bicistronic assays (3, 17). As with *Hoxa9*, many of the other previously reported *Hoxa* gene IRES-like TLs appear to be misannotated. For example, Xue et al. (3) defined a 1,106-nt TL for *Hoxa4* using 5′ RACE. However, the contemporaneous transcript annotation indicated a 15-nt leader, which is supported by RNA-seq data (*SI Appendix*, Fig. S11). Similarly, the 1,168- and 496-nt IRES-like TLs from mouse *Hoxa7* and *Hoxa11* appear to be only ∼112 and ∼90 nt long, respectively (*SI Appendix*, Fig. S10). Overall, our results suggest *Hoxa* mRNAs have shorter TLs translated via cap-dependent translation. Since it is much more efficient in developing embryos than cap-independent translation (58), cap-dependent translation would help ensure robust timely expression of these key developmental regulators.

Consistent with their misannotation, all the putative *Hoxa* IRESes we tested (*Hoxa3*, *Hoxa5*, *Hoxa7*, and *Hoxa11*) showed independent promoter activities, while non-IRES *Hoxa* TLs did not. Strikingly, sequences previously shown to be sufficient for putative *Hoxa3, Hoxa4, Hoxa5,* and *Hoxa11* IRES activities (3) overlap annotated promoters and TSSs (*SI Appendix*, Fig. S11). Moreover, conserved E-boxes were found in all the *Hoxa* TLs with putative IRESes (*SI Appendix*, Fig. S11), but not in non-IRES TLs. Mutating these E-box sites decreased the strength of *Hoxa* promoter activities. Depletion of *USF1* and *USF2* caused a significant, if modest, decrease in expression of the mouse and human *Hoxa9* reporters. However, mutating *Hoxa9* E-boxes had a stronger effect (Fig. 4), suggesting other bHLH transcription factors may also promote expression from these binding sites. Along those lines, other *Hoxa* reporters were not affected by *USF1*/*USF2* depletion, suggesting they may, instead, be regulated by other bHLH factors that recognize the same core motif (CACGTG) (33, 36). Notably, public mouse ChIP-seq data show that *USF1*, *USF2*, *MYC*, *MAX, TFE3*, *ARNTL*, *BHLHE40*, and *BHLHE41* bind to *Hoxa* gene E-boxes (*SI Appendix*, Fig. S7) (38–42, 44–51, 59–66). Several other E-box recognizing transcription factors, including *TCF15*, *HES1*, *HES7*, *MESP2*, and *MSGN1*, have been implicated in somite formation (67–72), and may also regulate *Hoxa* genes. More studies are needed to investigate the functions of conserved E-boxes in regulating *Hox* gene transcription during development.

Translational control of *Hox* genes was first suggested by a report that their translation was reduced in mouse embryos hemizygous for *RPL38* (*Ts/+*) (58). However, the data presented in that study do not actually show a decrease in Hox mRNA translation, typically seen as a shift from polysome to monosome sucrose gradient fractions. Instead, *Hox* mRNA were substantially reduced *in both* polysomes and monosomes in Ts/+ embryos, although the data were presented in separate figures (figures 3 and 6 in ref. 58). Furthermore, only a slight increase was observed in nontranslating fractions (58). Although the authors reported *Hox* gene mRNA levels were not decreased in *Ts/+* mutant embryos, the underlying RT-qPCR results had such high variance that even considerable changes in mRNA levels would be undetectable, and the *Hoxa9* primers used would not distinguish between mature mRNA, unspliced pre-mRNA, or fusion transcripts (Fig. 2). Even with optimal primers, RT-qPCR has several limitations in estimating mRNA levels (73–75). Additionally, a recent ribosome profiling study in HEK293 cells reported that depletion of *RPL38* decreased the translation efficiency of many genes that promote WNT signaling and *Hox* gene transcription (76). Future ribosome profiling studies from wild-type and Ts/+ embryos are needed to determine whether *RPL38* hemizygosity actually disrupts translation of *Hox* genes, their upstream transcriptional regulators, or both.

Our results also do not support the recently reported catalog of 589 hTLs in other mouse genes (17), 90 of which have putative IRESes based on bicistronic reporter assays. We showed these hTLs frequently overlap annotated promoters, enhancers, 3′ splice sites, and even protein CDSs, providing conventional explanations for their unusually high conservation rates. Furthermore, we tested four putative IRES regions from these hTLs and found that all encoded promoters. Indeed, the 90 IRES-like hTLs often show internal transcription initiation in public RNA-seq from ENCODE and 5′ CAGE-seq from RIKEN, and are particularly enriched in annotated promoters and splice sites, compared to non-IRES hTLs. We also used these features to build a model predicting bicistronic reporter activity. Notably, this model showed GC content and length were positive predictors of bicistronic activity—features that might appear consistent with structured IRESes. However, a high-throughput bicistronic IRES screen with controls to reduce promoter and splicing artifacts previously showed GC content was lower in active IRES elements (16), and high GC content is a known hallmark of promoter regions (55). Taken together, we propose that these hTLs are incorrect due to transcriptome annotation errors and promoter and splicing activities in bicistronic reporter assays. However, the concept of hyperconserved elements in 5′ TLs is still intriguing and deserves more careful study to identify genuine hTLs and investigate their functional elements.

It is well known that bicistronic reporter assays are subject to false positive results due to cryptic promoters and splice sites. Many control experiments have been devised to account for this. These include RNAi treatment to identify monocistronic *Fluc* transcripts, RT-PCR screening for cryptic splicing, and deletion of the SV40 promoter upstream of *Rluc* to account for independent promoter activities (7, 8, 20). Notably, a previous study of Hox gene IRES activity used siRNA targeting *Rluc* as a control for monocistronic transcripts. If only bicistronic transcripts were present, this treatment should deplete both *Rluc* and *Fluc* mRNA. Although siRNA treatment nearly eliminated *Rluc* mRNA, ∼30% of *Fluc* mRNA remained, consistent with monocistronic *Fluc* expression driven by promoter activities from the *Hoxa3*, *Hoxa4*, *Hoxa5*, *Hoxa9*, and *Hoxa11* IRES-like regions (3). Our results further support such monocistronic transcripts, as the putative IRES-like *Hoxa* TLs we tested had independent promoter activity, while non-IRES *Hoxa* TLs did not.

Previous work showed that the pRF plasmid has two cryptic promoters upstream of *Fluc* and *Rluc* which generate a variety of cryptic spliced products (*SI Appendix*, Fig. S1) (15). Using 5′ RACE, we identified *Rluc* transcripts containing multiple uORFs from one of these cryptic promoters, in the pMB1 origin of replication (*SI Appendix*, Fig. S5). We suspect the expression of *Rluc* by putative IRES test sequences (*SI Appendix*, Fig. S6) may reflect induction of other spurious transcripts, perhaps initiating at the other known cryptic promoter in the f1 origin of replication (15) (*SI Appendix*, Fig. S1). Regardless of the mechanism, the existence of these spurious transcripts further undermines comparisons of *Fluc*/*Rluc* protein and RNA ratios, previously used to discount cryptic promoters and splicing of putative IRES-like hTLs (17). These issues have been previously noted (7, 8, 15), with arguments specifically against using RT-qPCR for bicistronic assays, because it is unclear which transcripts are amplified in such assays (20).

Considering the cryptic promoters and splicing events associated with the pRF plasmid (15), IRES studies using this vector require rigorous controls (*Rluc* RNAi, promoter deletion, *Fluc* 5′ RACE) to eliminate the possibility of monocistronic transcripts from each test IRES sequence. However, it may be preferable to completely forego use of the bicistronic reporter. Because putative IRES sequences could alter the expression of spurious *Rluc* transcripts containing various numbers of uORFs (15) (*SI Appendix*, Figs. S1 and S5), which likely have variable mRNA stability and translation efficiency, *Rluc* mRNA and protein levels may also not be reliable as internal controls in the bicistronic reporter. Given these complications, we, instead, advocate testing IRES activity by comparing reporter expression from directly transfected m7G- and A-capped linear transcripts, using circular RNA reporters, or both (7, 8, 77, 78).

Our results underscore the importance of accurate transcript annotations for defining and studying TLs. The incorrect, extended *Hoxa9* TL can be traced to experiments that used reverse transcription “primer walking” to find the most upstream 5′ end (79). Unfortunately, this appears to have also amplified introns from *Hoxa9* fusion transcripts. Indeed, the region upstream of this misannotated extended TL is extremely G rich, such that G quadruplexes may have halted reverse transcription. As recently noted (56), the annotated mouse *Hoxa9* transcripts are 600 nt to 800 nt longer than expected given Northern blots in prior work (79, 80), further indicating their misannotation. However, such annotation errors are common, as many extended TLs from other genes also appear to be incorrect (e.g., *Hoxa4* and *Hoxa7*). In other cases, transcription initiates at multiple sites within annotated TLs (e.g., *Ptp4a1*, *Chrdl1, Dedd*). Astonishingly, even the TL of mouse *Actb* (beta actin) appears to be misannotated in RefGene, initiating with a TATA box and including a promoter (*SI Appendix*, Fig. S12). This error may explain why its TL showed apparent IRES-like activity when fused to the *Hoxa9* P4 domain (4). Similar errors may underlie additional putative IRESes from mRNAs that bind to ES9S in vitro (5), as most of these also include annotated promoter regions or other evidence of internal transcription initiation (*SI Appendix*, Fig. S9). Added to this is the general complexity of mammalian transcriptomes, in which TLs often include promoters, introns, and 3′ splice sites. Together, these issues make accurate mammalian TL annotation particularly challenging, and complicate the study of TL functional elements and conservation. Ongoing efforts to sequence full-length transcripts (81, 82), integrated with annotated promoters and TSSs, should eventually resolve such issues and greatly aid the study of TL functions in mammals.

## Materials and Methods

### Luciferase Vector Cloning.

The pRF+423Dux4 plasmid (Addgene #21625) contains Renilla Luciferase (*Rluc*) under the control of an SV40 promoter. *Fluc*, downstream of *Rluc*, is transcribed under the control of the same SV40 promoter and is preceded by a putative upstream IRES (*SI Appendix*, Fig. S1). The pRF+423Dux4 vector was sequenced using primers that anneal to the pGL3 vector (Promega; see primers in Dataset S2). The Dux4 IRES site was deleted from pRF+423Dux4 using PCR primers that flank the IRES region (PRF423DUX4-ATW F and R; Dataset S2). The primers also incorporated a BglII site after the start codon of *Fluc*, with an upstream HindIII site. The PCR product was phosphatased and circularized by ligation to create the vector pRF-ΔIRES. To delete the SV40 promoter, add an EcoRI site, and remove an additional BglII site, pRF-ΔIRES was used as a template for a second PCR, using the primers SV40D-EcoRI and SV40D-XhoI-R (Dataset S2). The resulting PCR product was phosphatased and circularized by ligation to create the vector pRF-ΔSV40. Both pRF-ΔIRES and pRF-ΔSV40 were verified by Sanger sequencing and tested for luciferase activity in C3H/10T1/2 mouse embryonic fibroblast (MEF) cells obtained from ATCC. An *Rluc* only vector was constructed by removing *Fluc* from pRF-ΔIRES by XbaI digestion. The XbaI cut vector was gel purified and circularized by ligation, resulting in the vector pR+SV40. The pR+SV40 vector was Sanger sequenced, and its luciferase activity was verified in MEF cells.

### Putative 5′ UTR Cloning.

Putative 5′ UTR sequences were obtained as double-stranded DNA fragments from Twist Biosciences and Genewiz (Dataset S2). The DNA fragments were PCR amplified, digested with HindIII and BglII, and cloned into the pRF-ΔSV40 vector at the HindIII and BglII sites upstream of *Fluc*. Due to limitations in DNA synthesis, five additional As were added by site-directed mutagenesis using MMHOXA11-ATW forward and reverse primers (Dataset S2) to finish the *Hoxa11* construct. Additional sequences in the *Hoxa3* hTL were removed by PCR using primers HOXA3-SATW forward and reverse primers (Dataset S2). Site-directed mutagenesis was also performed on Hoxa3, Hoxa5, Hoxa7, Hoxa9, and Hoxa11 constructs to mutate E-box sites to CACTAT. Some inserts affected both *Fluc* and *Rluc* (*SI Appendix*, Fig. S6), For more-precise ratiometric measurements, the *Rluc* gene was removed from wild-type and E-box mutant *Hoxa3*, *Hoxa5*, *Hoxa7*, *Hoxa11*, and *Hoxa9* constructs by EcoRI and HindIII digestion, end polishing with DNA polymerase I large fragment (Klenow), and religation (pF-ΔSV40; Fig. 4*C*). All constructs were Sanger sequenced (Dataset S2), and transfection-grade plasmid DNA was purified using a Qiagen Plasmid Mini column according to the manufacturer’s instructions.

### Luciferase Assays.

In 96-well tissue culture plates, 2 × 10^3^ MEF (C3H/10T1/2 clone 8, ATTC) cells were seeded in 100 μL of Dulbecco’s modified Eagle’s medium (DMEM) supplemented with 10% fetal bovine serum (FBS) per well. Cells were allowed to adhere and grow for 24 h at 37 °C. In 10 μL of Opti-MEM, 100 ng of construct was mixed with 0.4 μL of ViaFect (Promega) and incubated for 12 min at room temperature. The transfection mixture was added dropwise to the cells, and the cells were incubated at 37 °C for 24 h. *Fluc* and *Rluc* expression was assayed in a TECAN Spark plate reader using the Dual-Glo Luciferase Assay System (Promega) according to the manufacturer’s instructions. Both *Fluc* and *Rluc* were measured for 10 s per well.

### *USF1* and *USF2* siRNA Knockdown.

In a 96-well tissue culture plate, 1 × 10^3^ MEF (C3H/10T1/2 clone 8, ATTC) cells were seeded in 100 μL of DMEM supplemented with 10% FBS per well. Cells were allowed to adhere and grow for 24 h at 37 °C. In 10 μL of Opti-MEM, 1 pmol of siRNAs (scrambled control or USF1/2 siRNA; Santa Cruz Biotechnologies) were mixed with 0.3 μL of Lipofectamine 3000 transfection agent and incubated at room temperature for 15 min. The transfection mixture was added dropwise to the cells so that the final concentration of each siRNA was 10 nM. The cells were incubated at 37 °C for 24 h. For each well, 20 ng of pR+SV40 vector (Renilla only) and 80 ng of a Hox gene construct were mixed with 10 μL of Opti-MEM and 0.5 μL of ViaFect and incubated for 15 min at room temperature. The mixture was added dropwise to the cells, and the cells were incubated at 37 °C for 24 h. *Fluc* and *Rluc* were assayed as described above (*Luciferase Assays*).

### Validation of *USF1* and *USF2* siRNA Knockdown.

In a six-well tissue culture plate, 3 × 10^4^ C3H/10T1/2 cells were seeded in 2 mL of DMEM supplemented with 10% FBS per well. Cells were allowed to adhere and grow for 24 h at 37 °C. In 250 μL of Opti-MEM, 20 pmol of siRNAs (either scrambled control or USF1/USF2 siRNA) were mixed with 7.5 μL of Lipofectamine 3000 transfection agent and incubated at room temperature for 15 min. The transfection mixture was added dropwise to the cells so that the final concentration of each siRNA was 10 nM. The cells were incubated at 37 °C for 48 h. The media were removed, and total RNA was extracted using TRIzol (Invitrogen) following the manufacturer’s instructions. The total RNA was twice treated with TURBO DNase (Invitrogen) and purified over an RNA Clean and Concentrator-5 column (Zymo Research) after each DNase treatment. The RT-qPCR was performed in 50-μL reactions using the SuperScript III Platinum SYBR Green One-Step RT-qPCR kit (Invitrogen) with 200 ng of total RNA as template. Cycling and reaction conditions were followed according to the manufacturer’s instructions (Dataset S2 includes primer sequences). Three biological replicates were performed for the knockdown and scrambled control. Three technical replicates were performed for each gene, along with three technical replicates of no template controls. No amplification was detected for the no template controls. Relative gene expressions of USF1 and USF2 were compared to glyceraldehyde-3-phosphate dehydrogenase using the ΔΔ-Ct method (*SI Appendix*, Fig. S13).

### Logistic Regression.

We used active and nonactive hTLs provided by Gun Woo Byeon and Maria Barna (17). After removing records from TLs that were not previously classified as hyperconserved, the dataset included 133 nonactive hTLs and 90 active hTLs. We compiled a list of several categorical and numerical sequence features that could contribute to bicistronic activity (e.g., GC content, CAGE data, E-box motifs; Fig. 5 and Dataset S3). Transcript leaders lacking sufficient data for refTSS calculations (5′ refTSS CAGE reads) were assigned a mean imputation filler value. To perform classification of active versus nonactive transcript leaders, we used LogisticRegressionCV from scikit learn (sklearn.linear_model.LogisticRegressionCV) with the default solver = lbfgs, Cs = 10, intercept = True, and cv = 10 parameters. All numerical features were normalized using sklearn.preprocessing.MinMaxScaler. One hundred separate models were individually trained on random samples of 80% of the data and tested on the remaining 20% of the data.

### ENCODE RNA-seq Data.

From the ENCODE database, we used polyA plus RNA-seq data from *Mus musculus* and *Homo sapiens* tissues that expressed *Hoxa9*, assessed by visual examination. For the tissues containing multiple bigwig files, we merged the reads to create a new bigwig. The files containing “negative strand signal” data were used, because *Hoxa9* is on the negative strand. If no strand-specific file was given, then the “all reads” signal was used. For positive strand gene examples, the “positive strand signal” files were used. The accession numbers used are included in Dataset S4.

### CAGE Data (refTSS).

CAGE-seq data were downloaded using SRA Run Selector from National Center for Biotechnology Information from SRA study number DRP000949 (BioProject PRJDB1980). In this study, CAGE reads were obtained from Human and Mouse transcripts to define TSSs. For our study, we used the *M. musculus* data from runs DRR003905 (experiment DRX003141) and DRR003906 (experiment DRX003142). The data were from induced pluripotent stem cells and embryonic stem cells, respectively. Reads were processed using fastq-dump followed by cutadapt. The processed data were aligned to the mouse genome using STAR. Reads were summed and assigned to annotated refTSS peaks via bedtools intersect to define refTSS strength. Files used are listed in Dataset S4.

### Genome-Wide Information on Protein Synthesis RNA-seq Data.

RNA-seq data were retrieved from the Genome-Wide Information on Protein Synthesis (GWIPS) table browser (83) from Mouse (mm10) using the global aggregate setting. Data were compiled from 26 files (listed in Dataset S4). Bedgraphs were combined using bedtools unionbedg.

### Infernal and R-scape.

The sequence for the predicted mouse *Hoxa9* IRES from ref. 3 was used. Using the latest Zoonomia Cactus alignment file, we mapped the Mouse coordinates (chr6: 52226238 to 52226413) to 208 vertebrate species via halLiftover (84). An additional 23 vertebrate sequences were extracted from the University of California, Santa Cruz database for a total of 231 species. The sequences and results are in Dataset S1. The putative IRES structure from ref. 3 was converted into dot-bracket notation and used to generate a Stockholm format file containing the 231 sequences, the conserved structure, and the *M. musculus* sequence as reference.

The Infernal package was used to build a covariance model and prune the sequence alignment. Using default parameters for cmbuild and cmcalibrate, 25 close species were used to build and calibrate the initial covariance model. Using cmsearch, target sequences (230 sequences) with appropriate E values (default) for covariation testing were kept (medaka not significant, filtered out). A new Stockholm file was generated from the remaining sequences. The resulting file was used as input to R-scape using default parameters with various E-value thresholds (0.005, 0.01, 0.1, 1, 10, 20, and 30). The IRES data were tested seven times with the varying E values.

### The 5′ RACE from pRF Reporter Plasmids.

A total of 3.2 × 10^5^ C3H/10T1/2 cells (clone 8, ATTC) were seeded in 10 mL of DMEM (10% FBS) in 10-cm dishes and grown for 24 h at 37 °C. Seventeen micrograms of plasmid, 43 μL of Lipofectamine 3000, and 34 μL of P3000 reagent (ThermoFisher) were combined in 1 mL of Opti-MEM, incubated for 15 min at room temperature, and added dropwise to the cells. The cells were incubated at 37 °C for 24 h and harvested in 2 mL of TRIzol (Invitrogen). RNA was extracted following the manufacturer’s instructions. The RNA was pelleted by centrifugation at 20,000 × *g* for 30 min at 4 °C. The pellet was washed with 70% ethanol and resuspended in 200 μL of nuclease-free water. Forty-five micrograms of total RNA was twice treated with TURBO DNase (Invitrogen) and purified over an RNA Clean and Concentrator-25 column (Zymo Research). The RNA was eluted in 200 μL of nuclease-free water, and mRNA was selected using 75 μL of Oligo d(T)_25_ magnetic beads (New England Biolabs) according to the manufacturer’s instructions. Poly-A mRNA was eluted in 36 μL of nuclease-free water, and 12 μL was reverse transcribed in a 30-μL reaction using the Template Switching RT Enzyme Mix (New England Biolabs) according to the manufacturer’s instructions. Primers LUC-RT-R2 and Rluc-RT-R (Dataset S2) were used for F- and *Rluc*, respectively, and a mix of both primers was used in no-RT controls. The template switching oligo (TSO-Eno2; Dataset S2) adds a forward primer site for PCR. Complementary DNA was purified with AMPure XP magnetic PCR purification beads (Beckman Coulter), eluted in 10 μL of nuclease-free water, and PCR amplified for 35 cycles using the primers, ENO2LIBF1 and LUC-R for *Fluc* and ENO2LIBF1 and R-LUC-int-R for *Rluc*, in a 25-μL reaction with Phusion High-Fidelity DNA Polymerase (New England Biolabs) in high GC content buffer with dimethyl sulfoxide according to the manufacturer’s instructions. The PCR products were visualized on a TapeStation (Agilent). PCR products were electrophoresed on a 2% agarose gel, and visible bands were excised, gel extracted, and cloned using the Zero Blunt TOPO PCR cloning kit (Invitrogen) according to the manufacturer’s instructions.

## Supplementary Material

Supplementary File

Supplementary File

Supplementary File

Supplementary File

Supplementary File

## Data Availability

All study data are included in the article and/or supporting information.
