# Supplementary material for: False-positive IRESes from Hoxa9 and other genes resulting from errors in mammalian 5′ UTR annotations
Source: Proc Natl Acad Sci U S A. 2022 Aug 29;119(36):e2122170119. doi: 10.1073/pnas.2122170119 (PMC9456764; doi:10.1073/pnas.2122170119)
Supplement: Supplementary File [file pnas.2122170119.sapp.pdf]

PNAS
www.pnas.org

Supplementary Information for
False-Positive IRESes from *Hoxa9* and other genes resulting from errors in mammalian 5' UTR annotations

Christina Akirtava[a,1], Gemma E. May[a,1], C. Joel McManus[a,b,*].
[a]Department of Biological Sciences, [b]Computational Biology Department, Carnegie Mellon University, Pittsburgh PA 15213

*C. Joel McManus.
Email: mcmanus@andrew.cmu.edu

This PDF file includes:

Figures S1 to S13 (not allowed for Brief Reports)

Other supplementary materials for this manuscript include the following:

Datasets S1 to S4

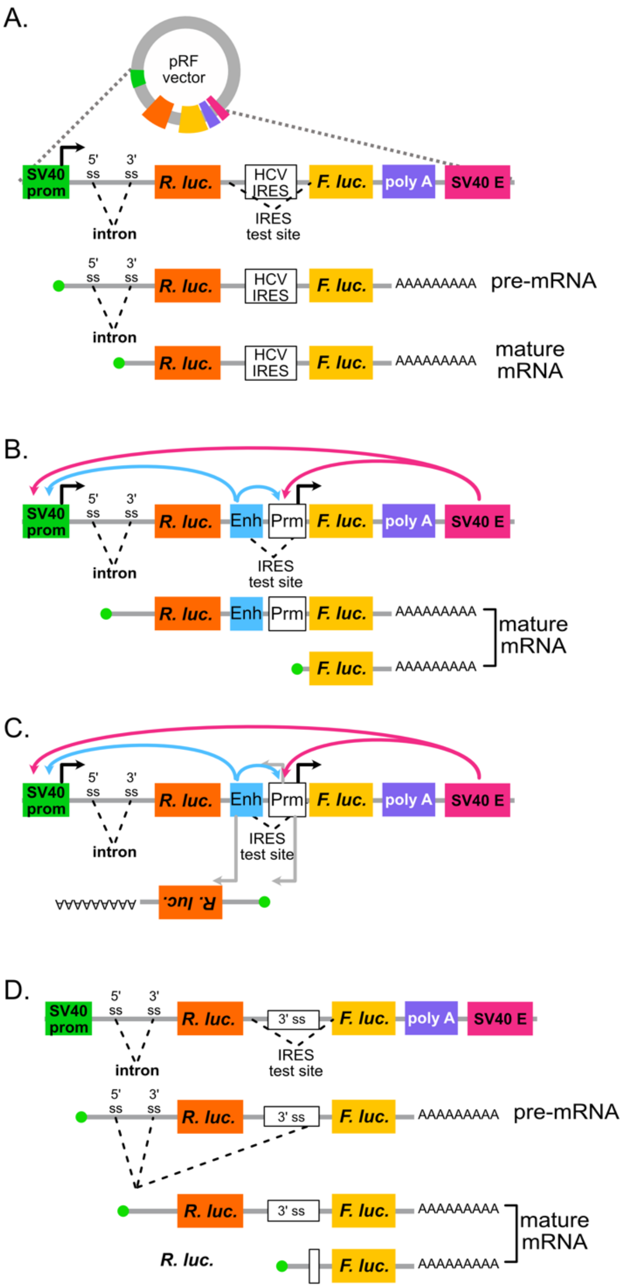

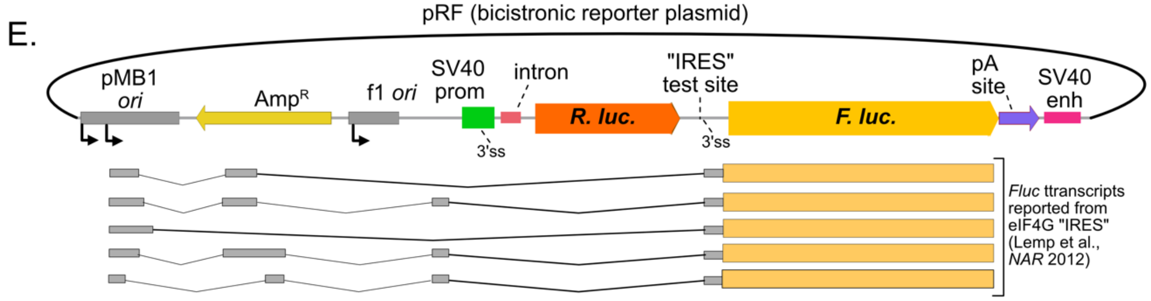

Figure S1. The pRF bicistronic reporter is subject to cryptic promoter and splicing activity. (A) Design of the pRF vector and expected mRNA products. Transcription is driven by the SV40 promoter (green), increased by an included SV40 enhancer (magenta). An intron is positioned upstream of Renilla luciferase (*Rluc*) to increase expression. An IRES test site between *Rluc* and Firefly luciferase (*Fluc*) ORFs is shown with the Hepatitis C Virus IRES (HCV). Transcription and splicing are expected to generate homogenous bicistronic transcripts. **(B)** Enhancer and promoter sequences placed in the IRES test site can generate monocistronic *Fluc* transcripts that give false-positive "IRES" signals. The "IRES" enhancer sequence (blue) can alter the transcription of both bicistronic and monocistronic transcripts. **(C)** The bidirectional transcription from promoters and enhancers may produce antisense *Rluc* transcripts, which would complicate qRT-PCR normalization attempts. (D) 3' splice sites (both strong and cryptic) in the IRES test site also create false-positive *Fluc* expression from monocistronic transcripts. **(E)** Lemp et al., (*NAR* 2012) showed the pMB1 and f1 replication origins have promoters that drive aberrant *Fluc* products via cryptic splicing. Arrows indicate transcription start sites. Aberrant transcripts from the eIF4G test "IRES" are shown. Cellular transcripts were also *trans*-spliced to *Fluc* in transfected tissue culture cells. Similar transcripts can affect *Rluc* (see Figure S5) and also leave additional *Rluc* RNA in spliced lariat introns. This likely undermines *Rluc* / *Fluc* qRT-PCR assays.

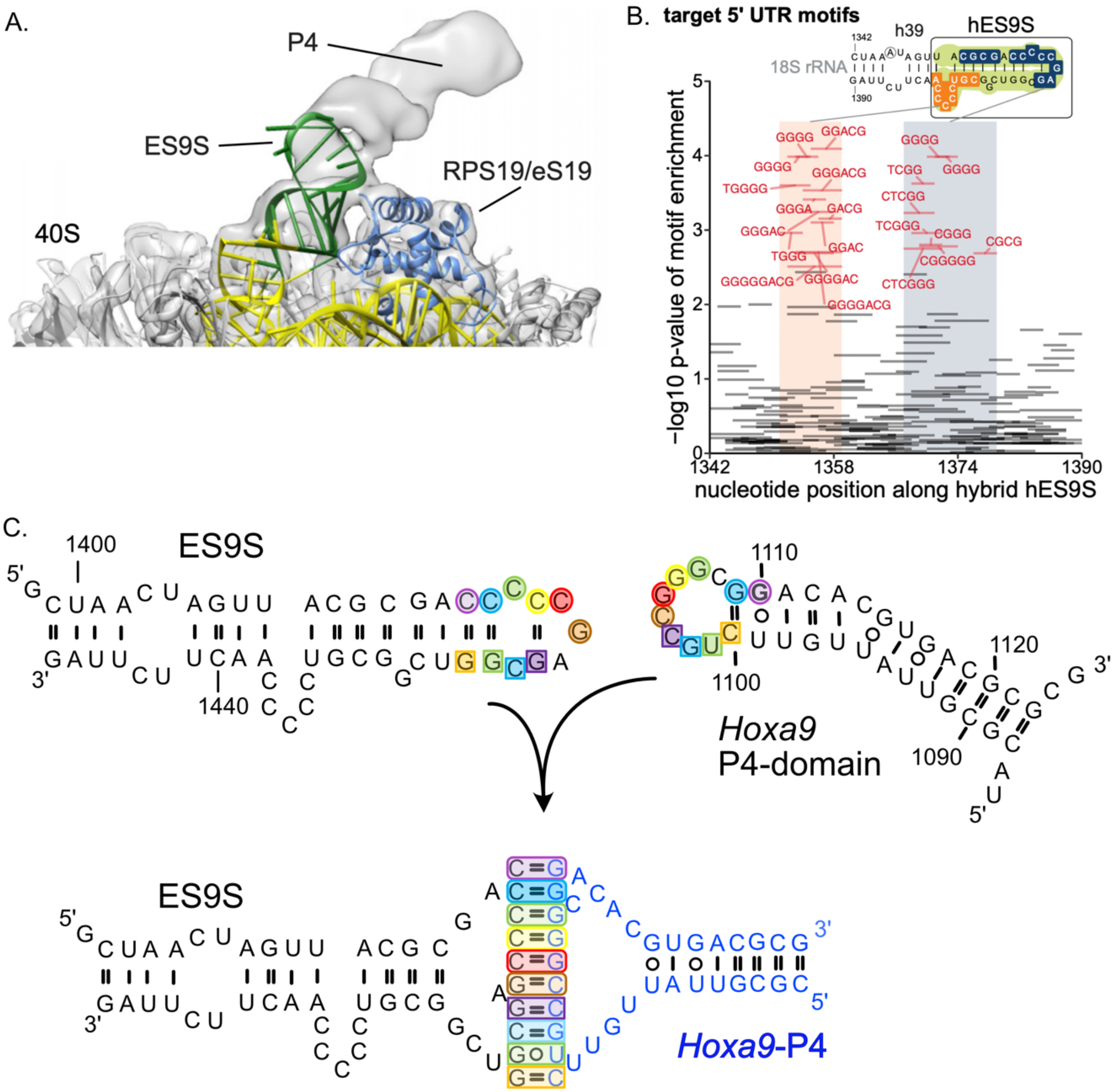

v

Figure S2. Models of interactions between ES9S, *Hoxa9* "P4" and mouse mRNA fragments. (A) The Cryo-EM interaction model published by Leppek et al., 2020. An A-form helix labelled "P4" appears oriented toward the C-rich loop of ES9S (green). (B) Leppek et al., 2021 figure panel 5a, showing G-rich motifs enriched in fragmented mouse mRNAs that bound to ES9S in vitro. The ES9S sequence was depicted above, with C-rich regions highlighted, as Leppek et al proposed these might form complementary Watson-crick pairs. (C) Mouse ES9S and putative *Hoxa9* IRES P4-domain have complementary sequences that could support a kissing stem loop interaction. Proposed structures of individual RNAs are depicted as reported from Leppek et al., 2020 and Xue et al., 2015 (above). Nucleotides that have the propensity to pair are shaded in matched colored ovals and squares. A G-rich segment in the putative *Hoxa9* IRES P4-domain is complementary to a C-rich segment in ES9S, with further potential pairing between additional adjacent nucleotides. Note the similarity to the interaction proposed by Leppek et al. 2021 (panel B) for ES9S binding to G-rich motifs in other mouse mRNAs (except *Hoxa9*).

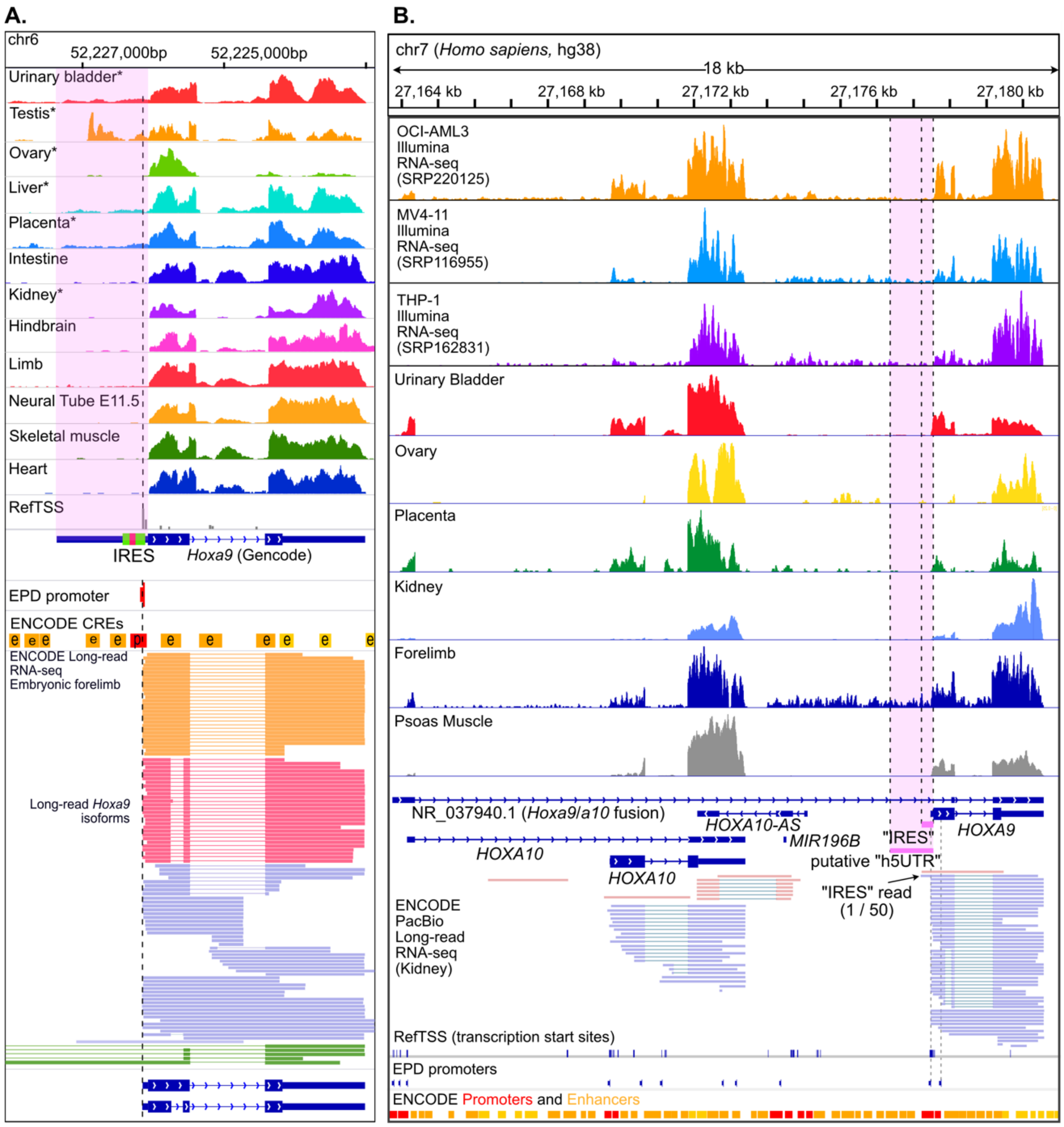

Figure S3. The putative IRES-like region of mouse and human *Hoxa9* are rarely expressed in mature transcripts. (A) ENCODE short- (upper) and long-read (PacBio, lower) RNA-seq data support transcription initiation almost exclusively downstream of the putative IRES-like region (shaded pink) in most mouse tissues, coinciding with an annotated promoter from EPD and ENCODE and 5' CAGE data and refTSS sites. Long-read data additionally suggest the extended isoform annotation may reflect intronic RNA from *Hoxa9*/*a10* and *Mir196b*/*Hoxa9* fusion transcripts (green). Asterisks denote strand-specific RNA-seq. (B) Human *Hoxa9* expresses short 5' UTR isoforms excluding the putative IRES. Genome browser tracks show short read polyA RNA-seq data from three Acute Myeloid Leukemia (AML) cell lines and data from the ENCODE project consortium from a variety of representative human tissues (upper). Long-read (PacBio Iso-seq) RNA-seq data from the ENCODE project (lower) shows two predominant isoforms whose transcripts initiate close to the *Hoxa9* protein coding sequence, consistent with annotated promoters (EPD and ENCODE) and transcription start sites (refTSS). One sense, and one antisense, Iso-seq read overlaps the putative IRES region.

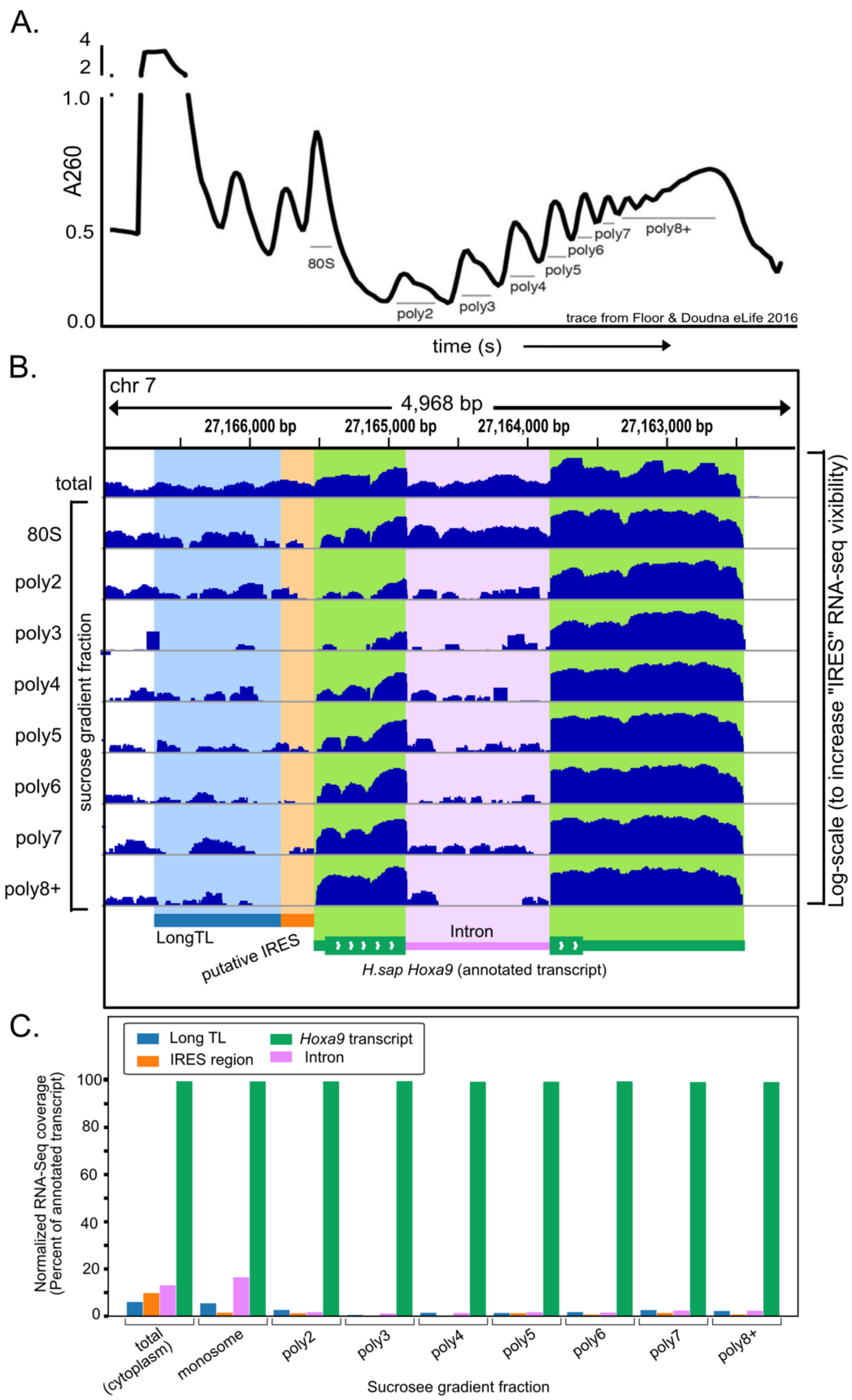

Figure S4. The extended 5' UTR and putative IRES are absent from translating *Hoxa9* mRNA in HEK293T cells. A) Polysome extracts from HEK293T cells were separated by sucrose gradient fractionation and RNA-seq was performed (Floor and Doudna, 2015). (B) IGV browser image shows RNA-seq coverage over four regions around human *Hoxa9*. A log-scale was used to increase the visibility of coverage over the putative IRES region. (C) The average coverage over each region is plotted, normalized to coverage over the annotated transcript. The extended 5' UTR and putative IRES regions have 5-10% the signal of the annotated transcript in total (ribo-depleted) RNA, which drops to ~ 1% in polysomal fractions. Both putative IRES and intronic RNA are almost entirely absent from translating polysome fractions (poly2 and greater).

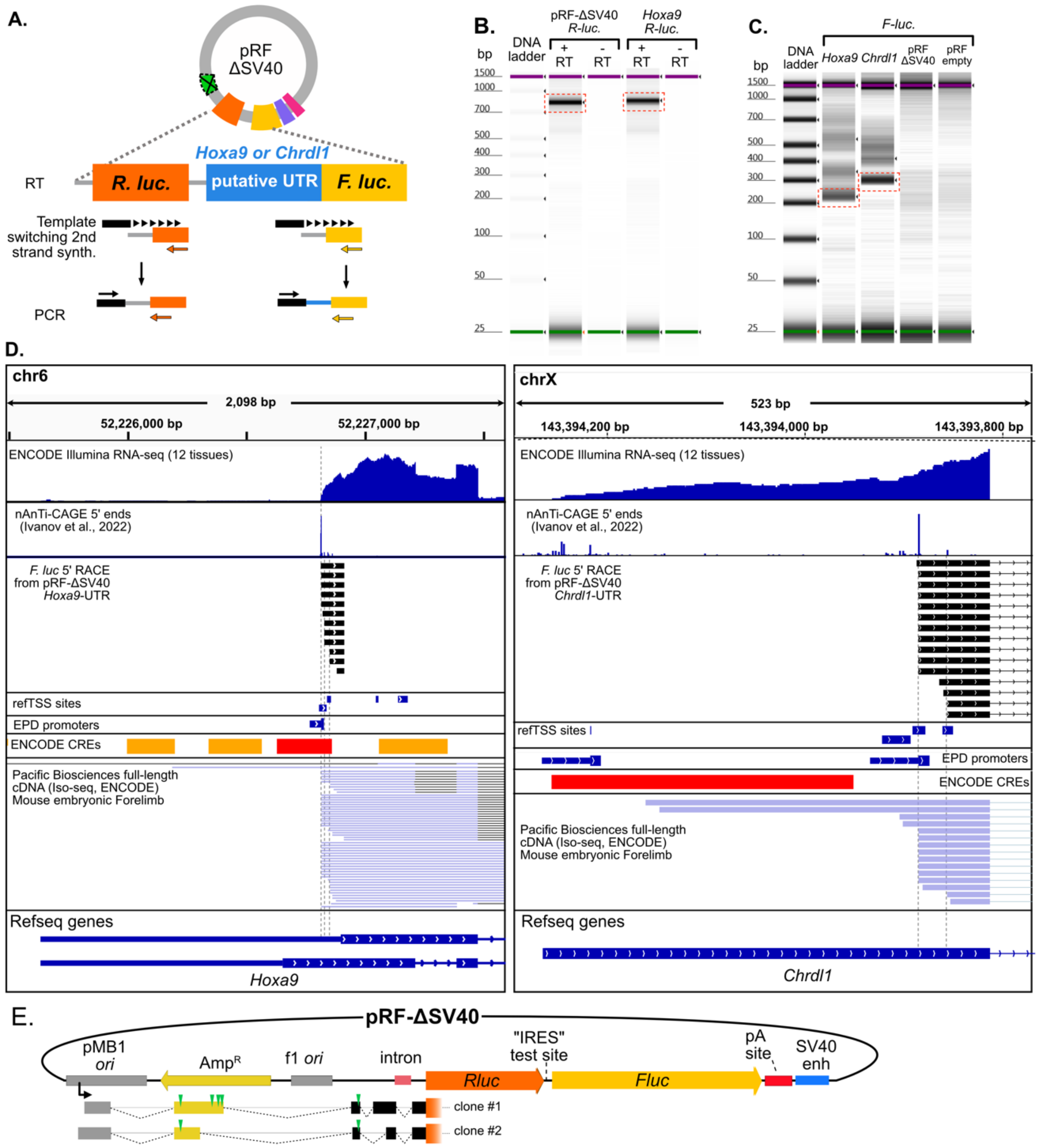

Figure S5. 5' RACE from pRF reporters recapitulates *in vivo* annotated *Hoxa9* and *Chrdl1*, TSS and identifies cryptic *Rluc* transcripts. (A) 5' RACE was performed on *Rluc*. and *Fluc* transcripts from C3H10T1/2 cells transfected with *Hoxa9* and *Chrdl1* 5' UTR pRF-ΔSV40 vectors. *Fluc* and *Rluc* RT and PCR primers are shown in yellow and orange, respectively, with the template switching oligo depicted in black. **(B)** *Rluc* RT-PCR products were electrophoresed on an Agilent TapeStation. A robust product (dotted red outlined box) was observed for both the *Hoxa9* 5'UTR and empty vector, despite the lack of an SV40 promotor. (**C)** *Fluc* RT-PCR electrophoresed as in B. Multiple short products (<700 bp) from *Hoxa9* and *Chrld1* putative UTRs, but not in empty-vector controls **(D)** The main RT-PCR products from *Hoxa9* and *Chrld1* transfections (C, red box) were cloned and sequenced. The cloned sequences, shown as genome browser tracks (black), recapitulate endogenous TSSs mapped with nAnTi-CAGE (Ivanov et al., 2022), and Iso-seq, from mouse embryonic tissues. **(E)** The RT-PCR products from *Rluc* (red box in B) were cloned and sequenced. Two clones are shown aligned to pRF-ΔSV40 with splice sites denoted with dotted black lines. These transcripts mapped to the pMB1 ori promoter (Lemp et al., 2012) and used many of the previously noted cryptic splice sites (Lemp et al., 2012). They are expected to have low translation efficiency due to multiple upstream AUG start codon uORFs (green triangles).

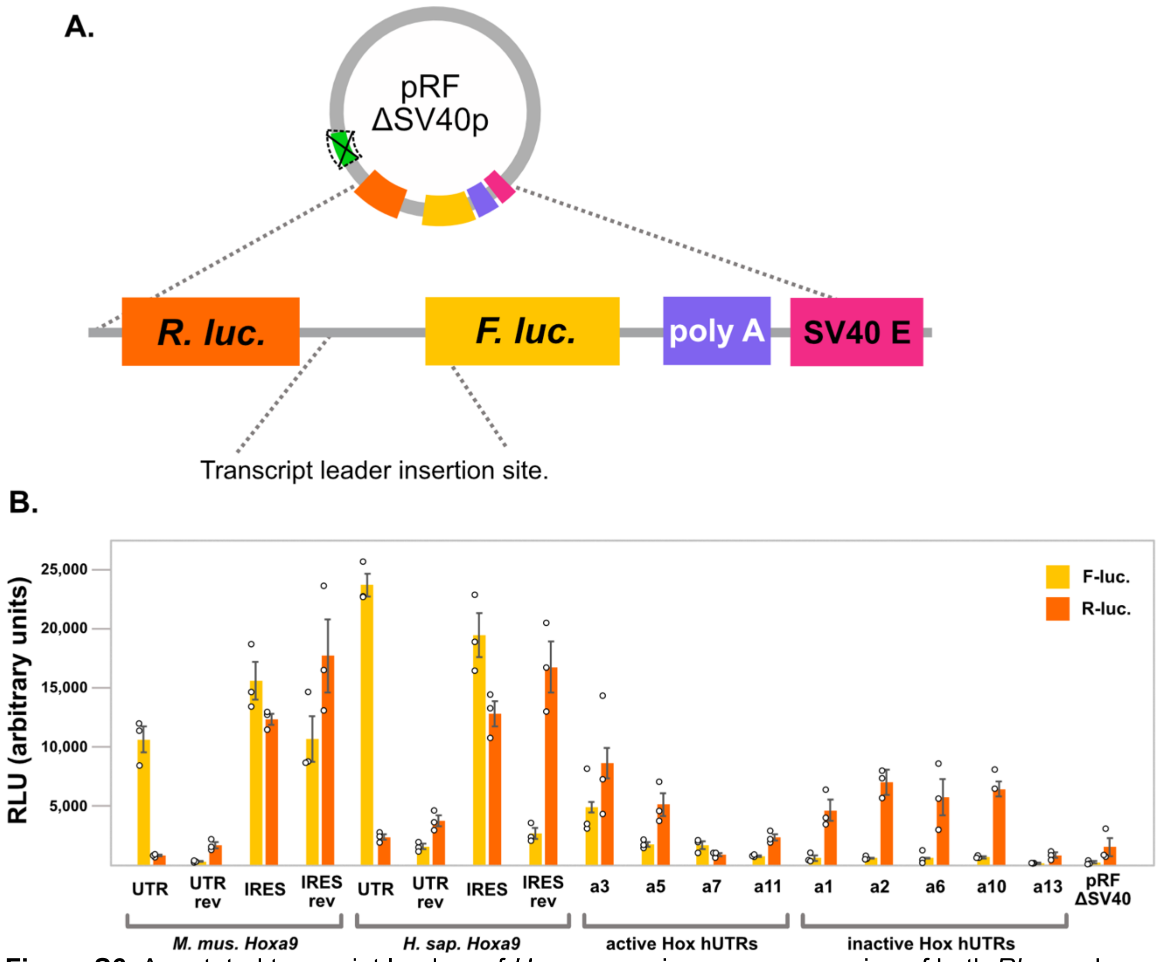

Figure S6. Annotated transcript leaders of *Hoxa* genes increase expression of both *Rluc* and *Fluc*. (A) Diagram of the "promoterless" bicistronic reporter plasmid pRF-ΔSV40. The putative IRES-like transcript leaders were cloned between *Renilla* (*Rluc*) and *Firefly* (*Fluc*) luciferase open reading frames and transfected into mouse mesenchymal cells. (B) Bar graphs showing raw luminescence values for *Rluc* and *Fluc* from each transfection. Error bars show standard error from three replicates. Most transcript leaders increase expression of both *Rluc* and *Fluc*, but to differing extents. For example, *M. mus. Hoxa9* UTR induces Fluc, but not *Rluc* expression well above background. The shorter "IRES-like" region induces expression of both, but Fluc is expressed more than *Rluc* compared to empty vector. The active (IRES-like) UTRs induce Fluc more than they induce *Rluc*, leading to a higher ratio (see figure 2), while the inactive UTRs induce similar fold changes in *Rluc* and *Fluc* expression, which the exception of *Hoxa13*, which induces neither gene. Note that this interpretation assumes does not account for potential variation in active plasmid concentrations during transfection. However, it seems unlikely that such variation could account for the variation in *Rluc* expression. Error bars show standard error with n = 3.

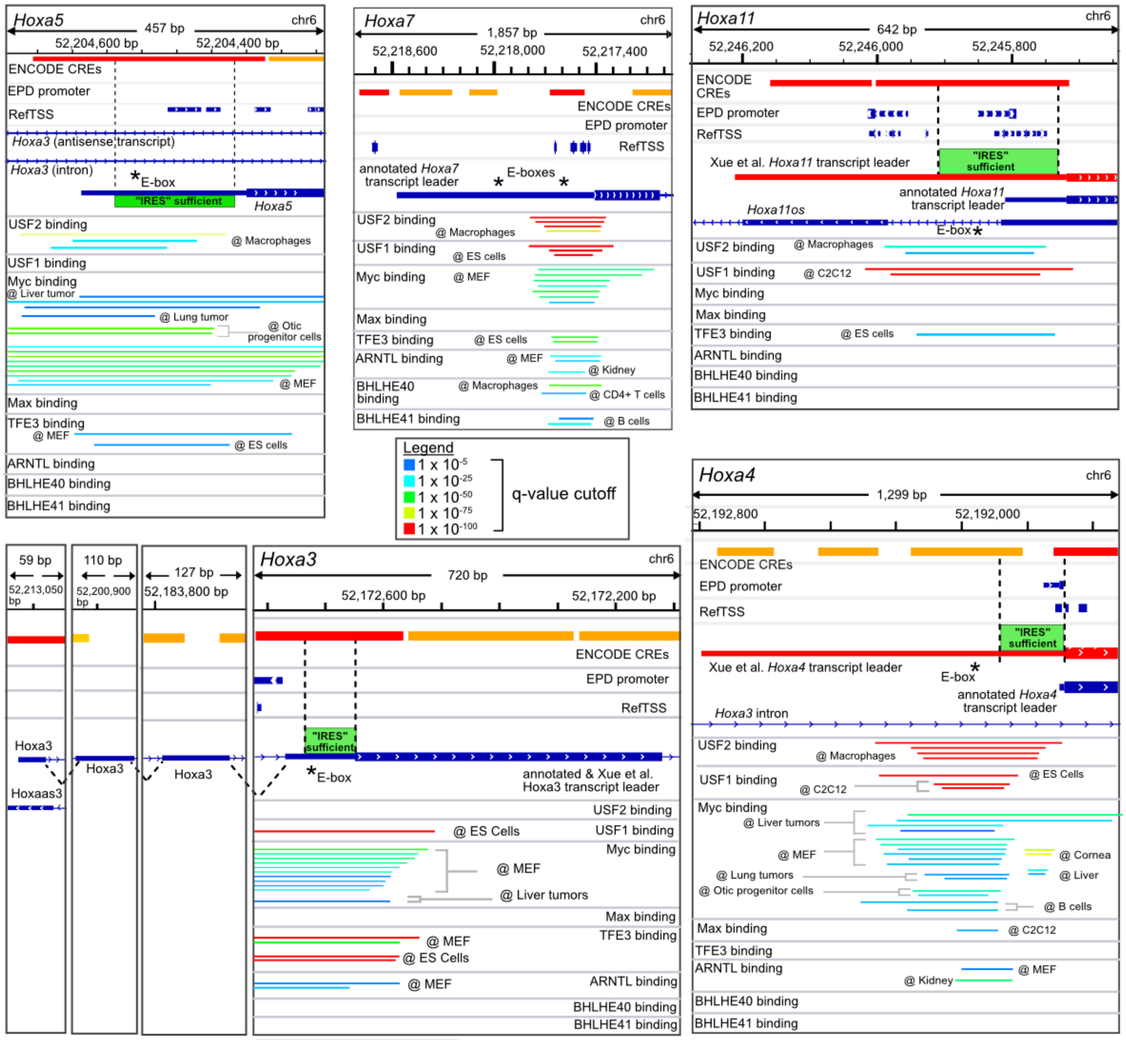

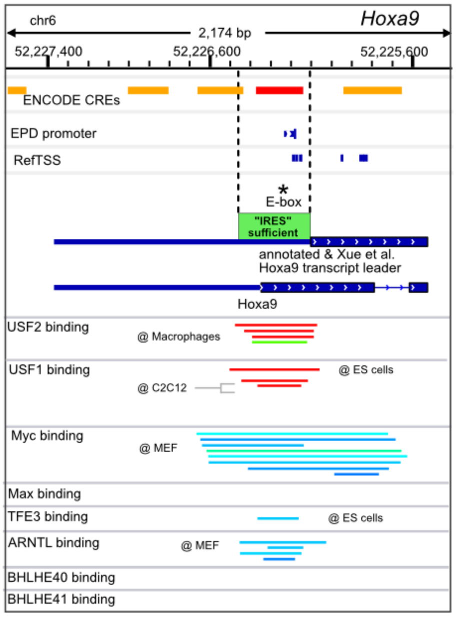

Figure S7. Mouse bHLH transcription factors bind to E-boxes near *Hoxa* gene promoters. Public mouse ChIP-seq peaks were downloaded from chip-atlas.org (Oki et al., *EMBO* 2018). Colored lines depict sites of significant binding, (MACS2 (v 2.10), see legend for FDR). Regions previously found to be important for "IRES" like activity in bicistronic reporter assays are shown above the gene annotation models. These regions overlap promoter elements annotated by ENCODE and / or EPD, and most contain annotated transcription start sites from RefTSS. In all cases, each *Hoxa* gene promoter region shows binding by at least one of bHLH transcription factor, with very strong binding of *USF1* and *USF2* in the "P4" element from the putative IRES region of *Hoxa9*. This is consistent with ChIP-seq studies from human *Hoxa9* and provides further evidence supporting these sequences as E-boxes. *Hoxa* E-box binding was also seen for other bHLH TFs, including *TFE3* (*Hoxa3*, a5, *a7,* d *a9, and a11*), *ARNTL* (*Hoxa3*, *a4*, *a7*, and *a9*), *BHLHE40* (*Hoxa7*), and *BHLHE41* (*Hoxa7*).

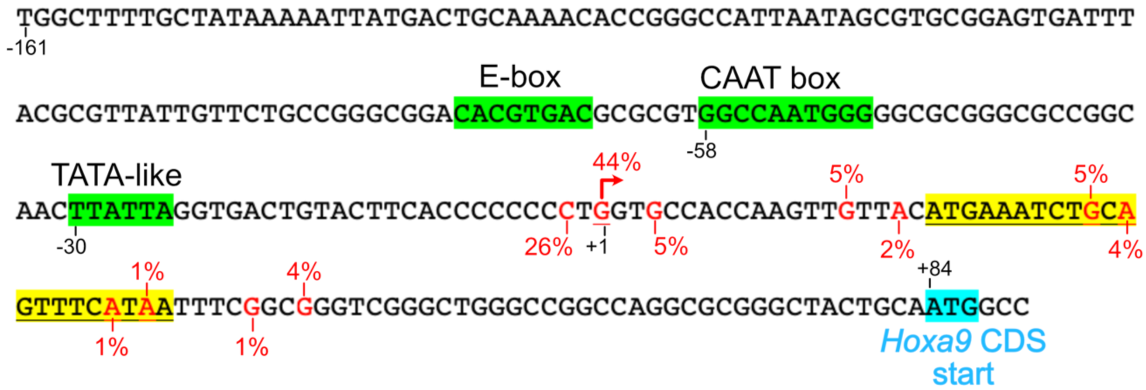

Figure S8. Sequence elements in the mouse *Hoxa9* promoter and 5' transcript leader. Sequence above shows the region upstream of mouse *Hoxa9*. The E-box, CAAT box, and TATA like elements are highlighted in green. Transcription start sites are shown in red text, with the percentage of nAnTiCAGE reads mapping to each position in mouse E11.5 somites (Ivanov et al., 2022). The major TSS site is indicated with an arrow. A conserved uORF with a poor Kozak context (Ivanov et al., 2022) is highlighted in yellow. The *Hoxa9* CDS start codon is shown in blue.

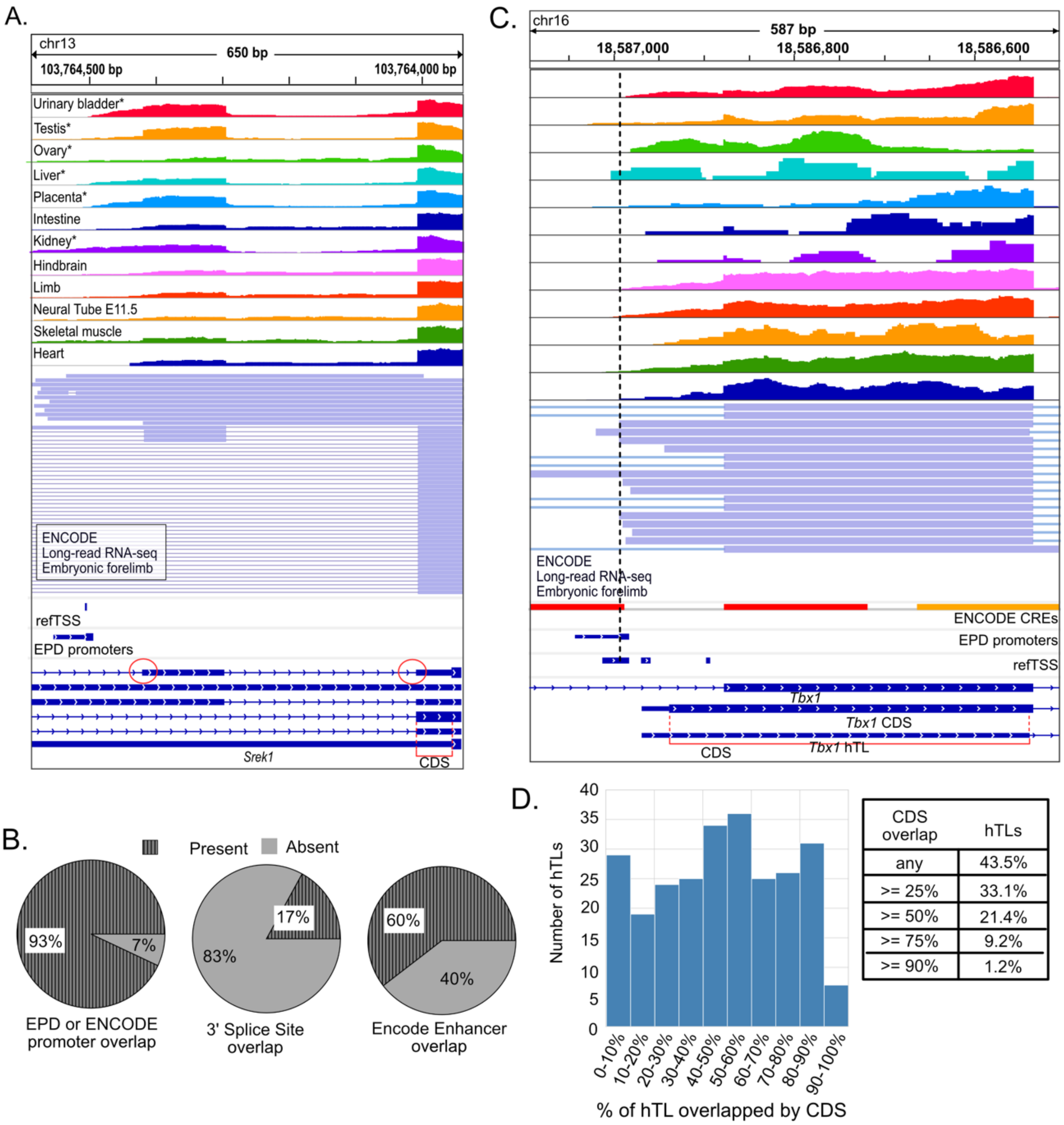

Figure S9. hTLs defined by Byeon et al. 2021 overlap promoters, splice sites, enhancers, and protein coding sequences. (A) Genome browser screenshot showing an example of a hTL from *Srek1*, which drives expression in the bicistronic reporter assay. Short- and Long-read RNA-seq suggest transcription initiates internally in this annotated transcript leader. The hTL overlaps an EPD promoter, two 3' splice sites, and protein coding sequence from an alternatively spliced isoform (CDS) of the gene. (B) Pie graphs showing the percentage of hTLs that overlap EPD or ENCODE promoters, annotated 3' splices sites, and ENCODE annotated transcriptional enhancer regions. (C) Genome browser screenshot showing a hTL from *Tbx1*, which almost entirely overlaps protein coding sequence from two other annotated transcript isoforms. The annotated protein coding sequence is translated in ribosome profiling data (GWIPs-VIZ; not shown) and has PhyloP conservation scores consistent with its translation (lower scores at wobble nucleotides). (D) Histogram showing the number of hTLs with varying percentages of CDS. 256 hTLs (43.5% of all hTLs) overlap annotated CDS regions, and a third of hTLs have at least 25% of their sequence overlapped with annotated CDS.

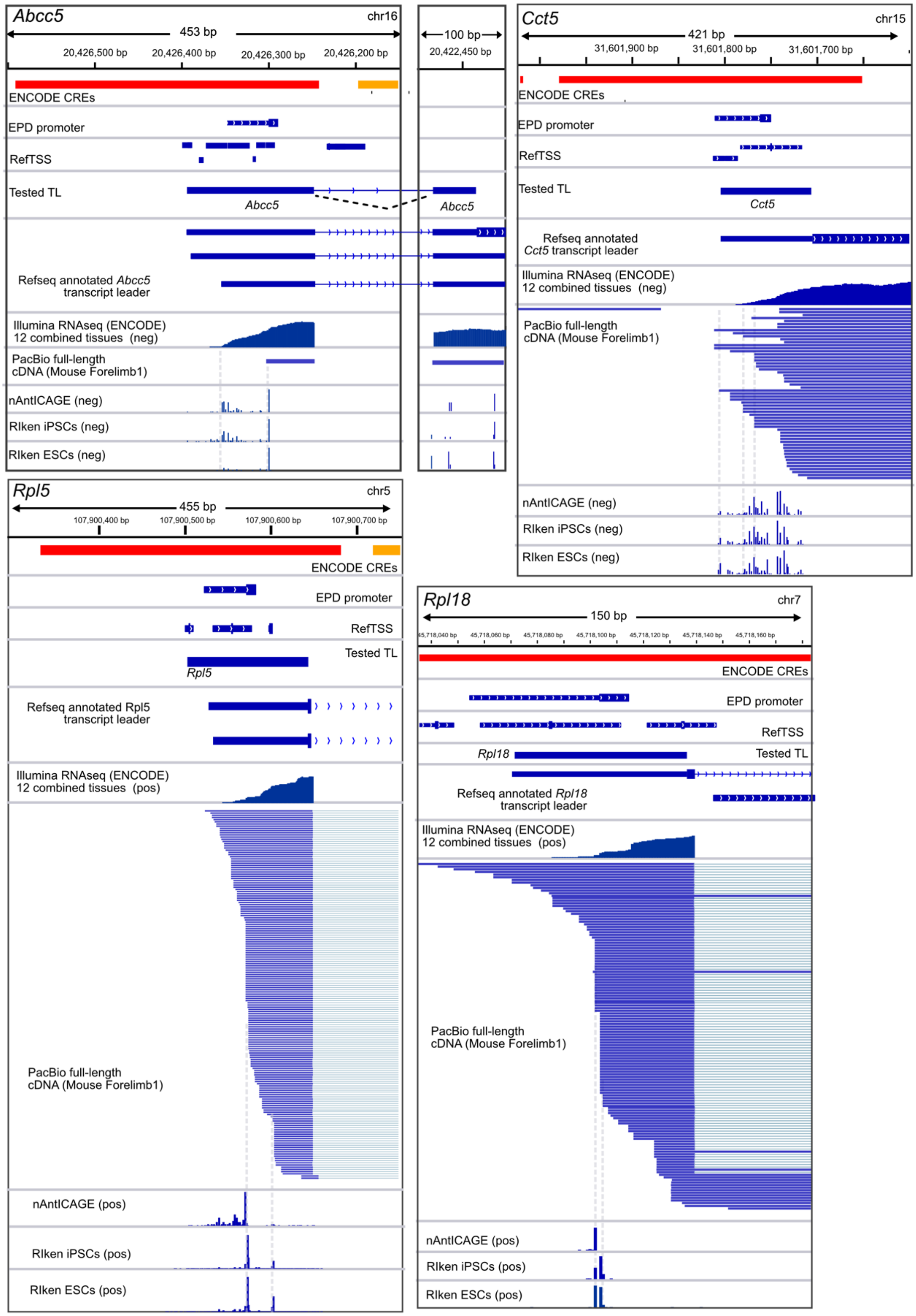

Figure S10. VELCRO-IP putative IRES elements contain annotated promoter sequences. Genome browser views show four putative IRESes from Leppek et al., 2021. EPD promoter elements are annotated within the putative IRES. PacBio Iso-seq and CAGE-seq data show numerous sites of internal initiation. Note, *Rpl5* was a "negative control", as it did not interact with the ES9S helix *in vitro*, yet it had strong bicistronic reporter activity.

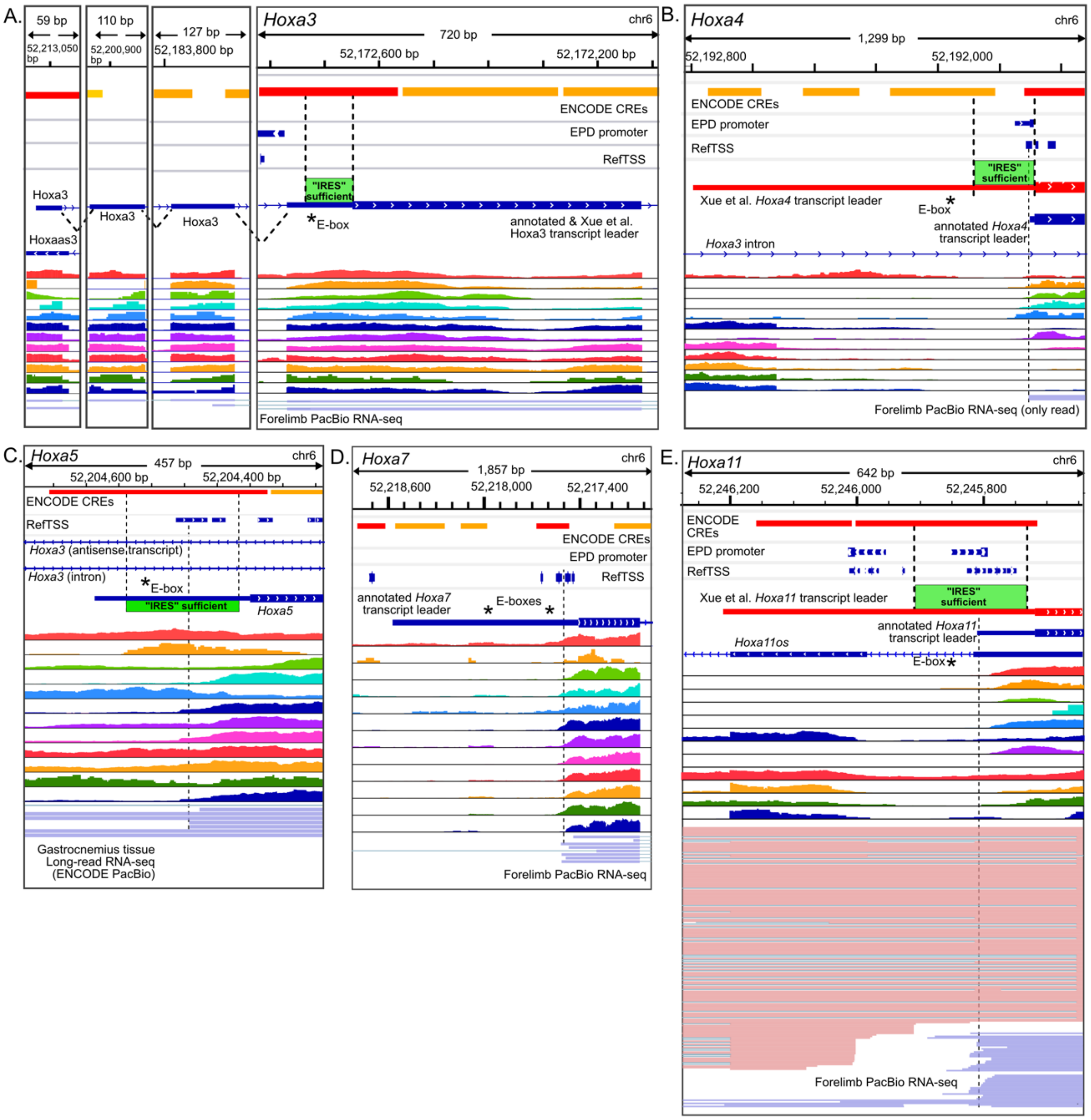

Figure S11. Misannotated transcript leaders and promoter overlap with putative IRESes from *Hoxa* genes. (A) The sufficient region of the putative *Hoxa3* IRES overlaps an ENCODE promoter. (B) The *Hoxa4* transcript leader mapped by Xue et al. 2015 is much longer than the annotated leader. The annotated, short leader is supported by short- and long-read RNA-seq data from ENCODE, and the sufficient region of the putative IRES overlaps an EPD promoter and refTSS sites. (C) The sufficient region of the putative *Hoxa5* IRES overlaps an ENCODE promoter and refTSS, which are supported by short- and long-read RNA-seq data. (D) The transcript leader of *Hoxa7* is much shorter than annotated, such that the region reported to be a putative IRES (Byeon et al., 2021) encompasses an ENCODE promoter and refTSS sites. (E) The *Hoxa11* transcript leader mapped by Xue et al. (2015) is much longer than the annotated transcript leader. The sufficient region of the putative IRES overlaps ENCODE and EPD promoters, and refTSS sites. Short- and long-read RNA-seq data support internal transcription initiation at the shorter annotated promoter. As with *Hoxa9* (Figure 2) extended, misannotated transcript leaders overlap introns in *Hoxa4*, and *Hoxa5*. * Asterisks show the locations of E-box motifs mutated in figure 3.

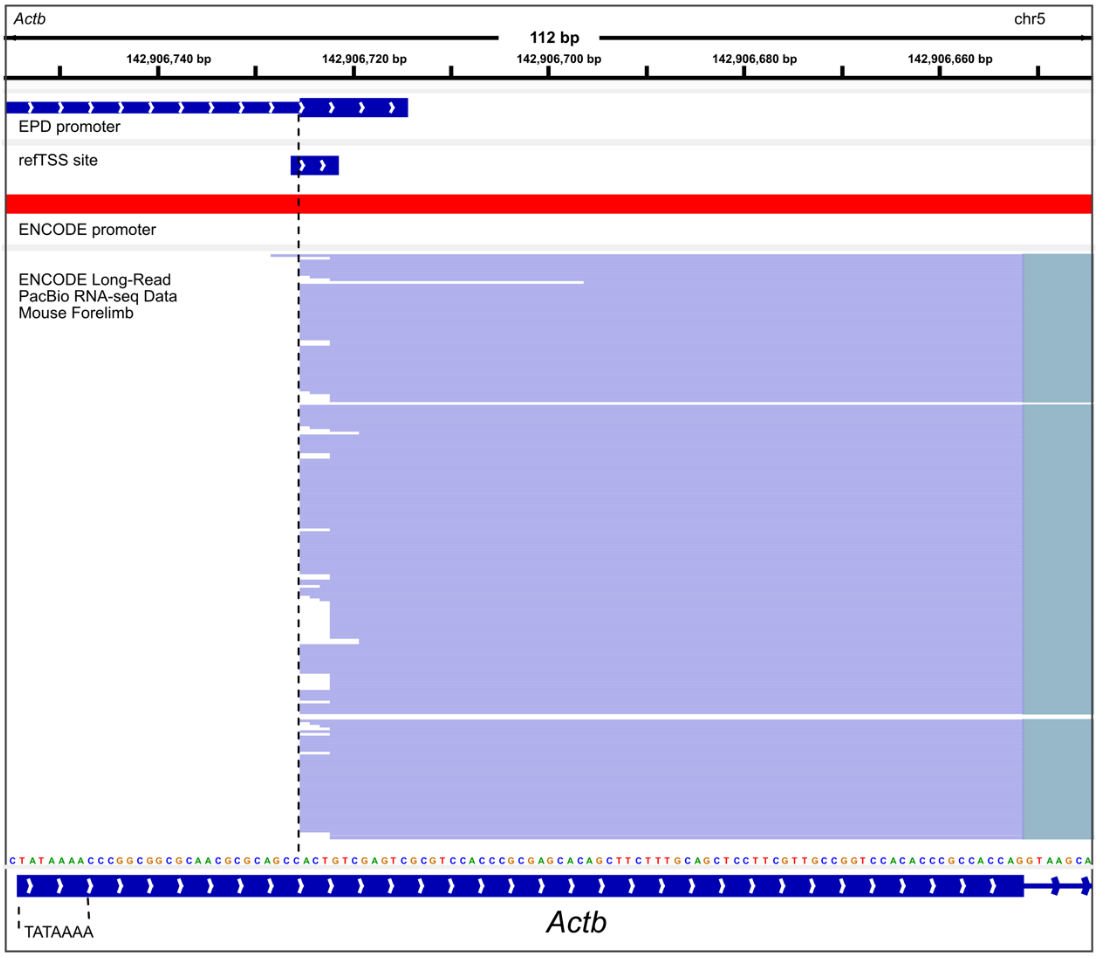

Figure S12. The transcript leader for mouse Beta actin is misannotated and includes a promoter. The refGene annotated transcript leader is shown. The annotated transcript leader begins with a TATA box, overlaps ENCODE and EPD promoters, and a refTSS site. Long-read RNA-seq data from ENCODE supports internal transcription initiation at the annotated promoter and refTSS site.

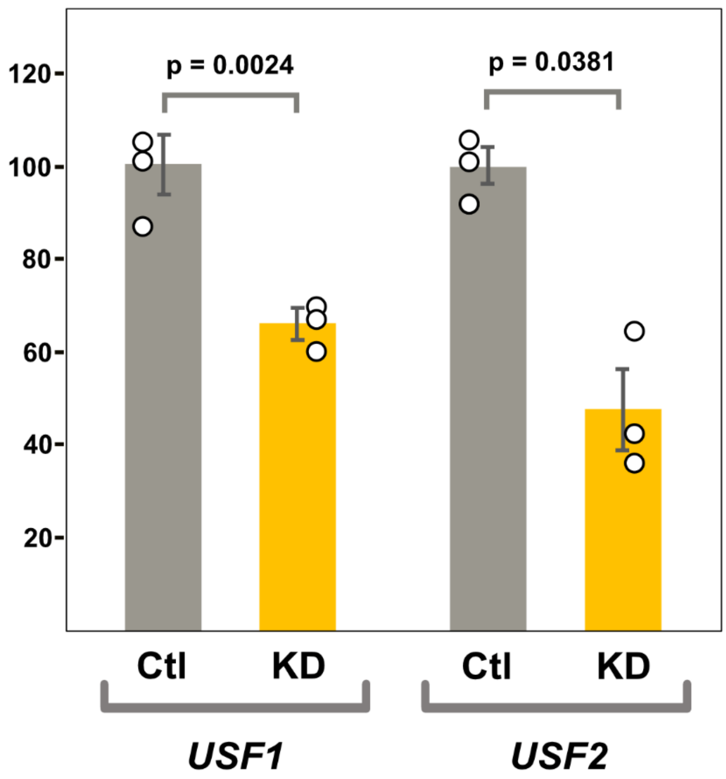

Figure S13. qPCR validation of *USF1*/*2* co-depletion by siRNA. *USF1* and *USF2* were assayed using qPCR to compare their mRNA levels from cells treated with control (scrambled) siRNA and cells treated with a mixture of *USF1* and *USF2* siRNA (Santa Cruz Biotechnology). Bar graphs show relative mRNA levels, normalized to control siRNA samples. Error bars indicate standard error. Both *USF1* and *USF2* mRNA levels were significantly depleted, compared to the scrambled control sample. P-values shown are from a 2-tailed, paired t-tests.

Dataset S1 (separate files). R-scape structural analysis. Dataset contains input sequences (FASTA), the mouse *Hoxa9* IRES predicted structure (MouseStructure), Rscape results (Rscape) and power analysis (Power).

Dataset S2 (separate file). Primer sequences used to clone reporter plasmids (Primers), Gene fragments ordered (Synthetic DNA Fragments), and Reporter insert sequences tested (Reporter Insert Sequences).

Dataset S3 (separate file). Features used for Logistic Regression Modeling.

Dataset S4 (separate file). RNA-seq and CAGE-seq data used in the study. Dataset includes the ENCODE file accession numbers and weblinks (ENCODE files) and the RIKEN CAGE-Seq data file accession numbers (CAGE-seq data).
